# Alcohol consumption and hepatocellular carcinoma: novel insights from a prospective cohort study and nonlinear Mendelian randomization analysis

**DOI:** 10.1186/s12916-022-02622-8

**Published:** 2022-10-28

**Authors:** Zhenqiu Liu, Ci Song, Chen Suo, Hong Fan, Tiejun Zhang, Li Jin, Xingdong Chen

**Affiliations:** 1 State Key Laboratory of Genetic Engineering, Human Phenome Institute, and School of Life Sciences, Fudan University, Shanghai, 200438 China; 2 Fudan University Taizhou Institute of Health Sciences, Taizhou, 225316 China; 3 Department of Epidemiology, School of Public Health, Nanjing Medical University, Nanjing, 211166 China; 4 Key Laboratory of Public Health Safety, Fudan University, Ministry of Education, Shanghai, 200032 China; 5 Department of Epidemiology, School of Public Health, Fudan University, Shanghai, 200032 China; 6 National Clinical Research Center for Aging and Medicine, Huashan Hospital, Fudan University, Shanghai, 200040 China

**Keywords:** Alcohol intake, HCC, Wine, Cohort study, Mendelian randomization

## Abstract

**Background:**

Heavy drinking was well associated with an increased risk of hepatocellular carcinoma (HCC), whereas the effect of low-to-moderate drinking on HCC remains under debate.

**Methods:**

Participants from the UK Biobank with detailed information on alcohol use and free of common diseases were included. Daily pure alcohol intake (g/day) was calculated, and the predominant alcoholic beverage type was assigned for each participant. Additive Cox regression model and nonlinear Mendelian randomization (NLMR) analyses were performed to evaluate the association of alcohol intake with HCC.

**Results:**

Of 329,164 participants (52.3% females, mean [SD] age = 56.7 [8.0] years), 201 incident HCC cases were recorded during the median follow-up of 12.6 years. The best-fitted Cox regression model suggested a J-shaped relationship between daily alcohol intake level and HCC risk. However, NLMR analysis did not detect a nonlinear correlation between alcohol use and HCC (nonlinearity *P*-value: 0.386). The J-shaped correlation pattern was detected only in subjects who mainly drank wine but not in those who mainly drank beer, spirits, or fortified wine. Moderate wine drinking showed a significant alanine transaminase (ALT)- and aspartate aminotransferase-lowering effect compared to that of the nondrinkers. In low-risk populations of HCC including women, people aged < 60 years, subjects with normal ALT levels, and those carrying non-risk genotypes of *PNPLA3* rs738409 and *TM6SF2* rs58542926, we observed a J-shaped correlation between alcohol use and HCC; however, a positive dose–response correlation was found in their respective counterparts, even in those predominantly drinking wine.

**Conclusions:**

Low-to-moderate drinking may be inversely associated with the risk of HCC in low-risk populations, which may be largely driven by wine drinking. However, those in high-risk populations of HCC, such as men and older people, and those with abnormal ALT levels and carry genetic risk variants, should abstain from drinking alcohol. Given the small HCC case number, further validations with larger case numbers are warranted in future works.

**Supplementary Information:**

The online version contains supplementary material available at 10.1186/s12916-022-02622-8.

## Background

Hepatocellular carcinoma (HCC) accounts for the great majority of liver cancer diagnoses and deaths and has shown an increasing disease burden worldwide in recent decades [1–3]. Previous studies showed that the risk of liver cancer proportionally increased with the amount of alcohol consumption [4, 5], suggesting that there is no safe level of alcohol consumption for liver cancer [6]. For example, in a cohort study of nearly 0.2 million Chinese men, there was a positive dose–response relationship between the amount of alcohol intake and the risk of liver cancer [7]. However, several studies reported that low-to-moderate alcohol drinking was inversely associated or not significantly associated with the risk of liver cancer (or HCC) [8–10]. For example, a meta-analysis of prospective studies reported a suggestive but not statistically significant decreased risk of liver cancer in individuals with moderate drinking (< 3 drinks per day) compared with abstainers [10]. Findings from the Liver Cancer Pooling Project showed that light-to-moderate drinking appeared to be inversely associated with HCC risk [8].

The inconsistencies among observational studies may ascribe to many reasons, including different study designs, small case numbers, incomplete adjustment for confounding factors, reverse causation, and failure to measure lifetime use and patterns of alcohol intake [11]. Moreover, misclassifying prior heavy drinkers who abstain from drinking currently and nondrinkers who suffer from chronic diseases, such as cardiovascular diseases and diabetes, as abstinence may lead to a spuriously increased risk of liver diseases in this population. Alcoholic beverage type may also affect the association of alcohol intake with HCC [8]. Additionally, genetic factors that are closely linked to liver diseases may modify the association between alcohol use and liver diseases but were rarely considered in previous studies [12].

To fill these gaps, in the current study, we dissected the correlation between alcohol consumption and the risk of HCC among 329 thousand UK Biobank participants with the full considerations of potential limitations. In addition, we evaluated whether the associations varied by age, sex, alanine transaminase (ALT) levels, alcohol type and intake frequency, or genetic variants of *PNPLA3* and *TM6SF2*.

## Methods

### Study participants

This research was conducted using the UK Biobank Resource under application number 63726. In this study, we retrieved demographic (e.g., age and sex), epidemiological (e.g., alcohol intake), laboratory (e.g., ALT and aspartate aminotransferase [AST]), and genetic data. Participants who were missing > 30% of laboratory data (*n* = 1567), were incalculable for alcohol intake volume (*n* = 103,218), had a daily pure alcohol intake > 200 g (*n* = 443), were not of Caucasian ethnic background (*n* = 21,695), or suffered from chronic diseases that may influence alcohol drinking behavior (*n* = 46,418) were excluded (Additional file 1: Fig. S1). Finally, a total of 329,164 participants, among which 12,342 were lifetime abstainers and 316,822 were current drinkers, were included.

### Pure alcohol intake calculation

Pure total alcohol intake in grams was calculated by multiplying the average number of alcoholic drinks consumed each week/month by the average grams of alcohol contained in each type of drink (Additional file 2: Table S1) [13]. It was then divided by seven or thirty to calculate daily mean alcohol intake (g/day). We defined participants who reported never drinking, previously or currently, as lifetime nondrinkers. We excluded participants who drank ≥ 200 g/day to prevent outliers from having undue leverage in the analyses. Men with daily < 30 g, 30–60 g, and > 60 g pure alcohol intake were defined as low-to-moderate, excess, and heavy drinkers, respectively. Women with daily < 20 g, 20–40 g, and > 40 g pure alcohol intake were defined as low-to-moderate, excess, and heavy drinkers, respectively. To identify the alcohol intake pattern, we calculated the intake volume of pure alcohol from wine, spirits and fortified wine, beer, and other types of alcohol beverages. The dominant beverage type of each participant was defined as a beverage contributing ≥ 75% of the total pure alcohol volume; otherwise, no dominant type of beverage was assigned. Alcohol intake frequency was also retrieved and categorized into four groups: never, less-frequent, frequent, and regular (Table 1).

**Table 1:** Numbers and incidence rates of hepatocellular carcinoma among the UK biobank participants

Characteristics	*N* (%)	No. of HCC cases	Incidence rate of HCC (per 100,000)	*P* value^†^
Sample size	329,164 (100)	201	4.83	
Sex				< 0.001
Male	157,043 (47.7)	154	7.09	
Female	172,121 (52.3)	47	2.36	
Age (years)				< 0.001
< 60	186,567 (56.7)	51	2.84	
≥ 60	142,597 (43.3)	150	6.34	
Alcohol intake level				< 0.001
Abstinent	12,342 (3.7)	7	4.46	
Low-to-moderate	190,180 (57.8)	91	3.80	
Excess	89,012 (27.0)	42	3.71	
Heavy	37,630 (11.4)	61	12.73	
Alcohol intake frequency^c^				< 0.001
Never	12,342 (3.7)	7	4.46	
Less frequent	130,745 (39.7)	60	3.66	
Frequent	99,606 (30.3)	59	4.66	
Regular	86,471 (26.3)	75	6.83	
Dominated alcohol type^b^				< 0.001
Wine	82,038 (24.9)	37	3.57	
Beer	51,495 (15.6)	51	7.84	
Spirits and fortified wine	11,597 (3.5)	8	5.44	
Non-specific	171,692 (52.2)	98	4.51	
Usually drinking with meal				< 0.001
Yes	139,358 (44.0)	64	3.64	
No	63,981 (20.2)	53	6.53	
Varies	113,483 (35.8)	77	5.35	
Smoking status				< 0.001
Never	179,851 (54.6)	69	3.03	
Former	116,634 (35.4)	104	7.05	
Current	32,679 (9.9)	28	6.75	
Average household income				< 0.001
Less than 18,000	50,569 (15.4)	50	7.84	
18,000 to 30,999	69,623 (21.2)	56	6.37	
31,000 to 51,999	122,001 (37.1)	67	4.33	
52,000 to 100,000	67,829 (20.6)	21	2.45	
Greater than 100,000	19,142 (5.8)	7	2.91	
Physical activity				< 0.001
0–1 day/week	62,965 (19.1)	40	5.01	
2–4 days/week	141,931 (43.1)	88	4.90	
5–7 days/week	124,268 (37.8)	73	4.65	
BMI category				< 0.001
< 25	116,960 (35.5)	36	2.43	
25–29.9	144,652 (43.9)	81	4.43	
> = 30	67,552 (20.5)	84	9.84	
*PNPLA3* rs738409^a^				< 0.001
CC	197,439 (61.5)	97	3.88	
CG/GG	123,662 (38.5)	102	6.53	
*TM6SF2* rs58542926^a^				< 0.001
CC	274,776 (85.6)	152	4.37	
CT/TT	46,325 (14.4)	47	8.04	
ALT level (U/L)				< 0.001
< 40	303,691 (92.3)	114	2.97	
≥ 40	25,473 (7.7)	87	26.90	
HBV/HCV status^a^^,d^				0.876
Positive	116 (1.9)	0	0	
Negative	6040 (98.1)	1	1.31	

*BMI* Body-mass index, *ALT* Alanine aminotransferase, *HBV* Hepatitis B virus, *HCV* Hepatitis C virus

^†^*P* values were calculated from the likelihood ratio test in univariate Cox regression model

^a^8063 individuals were missing on genetic data; 323,008 individuals were missing on HBV/HCV status

^b^The dominated beverage type of each participant was defined as the beverage contributing ≥ 75% to the total pure alcohol volume. Otherwise, no dominated type of beverage was assigned

^c^Never was defined as life-time nondrinkers; less-frequent defined as drinking less than twice per week; frequent defined as drinking three or four times per week; regular defined as drinking more than five times per week

^d^HBV seropositivity defined as both antigen HBc > 100 and antigen HBe > 150; HCV seropositivity defined as both antigen Core > 150 and antigen NS3 > 150

### Outcome definition

We used the International Classification of Disease version 10 (ICD-10) code C22.0 to identify incident HCC. We identified the prevalent diseases at baseline using both ICD-10 and ICD-9 codes (Additional file 2: Table S2). Participants who had any type of liver disease were excluded. We also excluded participants with a history of chronic diseases that may influence alcohol drinking behavior (e.g., type 2 diabetes and hypertension) (Additional file 1: Fig. S1).

### Genotype data

The genotype data in the UK Biobank were derived from the GWAS chip (Affymetrix UK BiLEVE and UK Biobank Axiom arrays). In this study, we selected two genetic variants, *PNPLA3* rs738409 C/G and *TM6SF2* rs58542926 C/T, which are the most robustly replicated SNPs that are well-associated with alcoholic liver diseases [14, 15]. We used the genotypes G of rs738409 and T of rs58542926 as the risk alleles.

### Covariates

We retrieved information on age at enrolment, sex, average annual household income, smoking status, physical activity level, education deprivation score, body-mass index (BMI), and biochemical indices. Smoking status was classified as never, former, or current. Average annual household income was categorized into five groups: < £18,000, £18,000–30,999, £31,000–51,999, £52,000–100,000, and > £100,000. We used the number of days per week of moderate physical activity that lasted for ≥ 10 min as a surrogate to measure physical activity level. Three groups were formed for physical activity level: 0–1 day/week, 2–4 days/week, and 5–7 days/week. Education deprivation score is a composite index measuring the extent of deprivation in terms of education, skills, and training in an area. For epidemiological covariates, we applied median value imputations for the missing values.

### Statistical analyses

We calculated the HCC incidence rate (per 100,000 person-years) for subgroups. To assess the curvilinear association between alcohol intake and HCC, we incorporated nonlinear effects of alcohol intake into the Cox regression model to express the log hazard as an additive function (Additional file 3: Method S1) [16]. In this analysis, the alcohol intake was fitted in a continuous form and was represented using natural cubic splines with the degree of freedom (*df*) selected by minimizing Akaike’s Information Criterion (AIC). To overcome overfitting, we set *df* to range from 1 to 5. Intercept was not included in the basis; therefore, *df* = 1 denotes a linear relationship. In the additive Cox regression model, we adjusted for age, sex, smoking status, physical activity level, education deprivation score, household income, and BMI. The hazard ratio (HR) and its 95% confidence interval (CI) were calculated to quantify the association. Proportional hazards assumptions for Cox regression models were tested. The additive Cox model was also performed in subgroups by age (< 60 and ≥ 60 years), sex, baseline ALT levels (< 40 and ≥ 40 U/L), alcohol beverage types (wine, beer, spirits and fortified wine), alcohol intake frequency (less-frequent, frequent, and regular), alcohol usually consumed with a meal (yes, no), and genotypes of *PNPLA3* rs738409 (CC, CG/GG) and *TM6SF2* rs58542926 (CC, CT/TT).

### Nonlinear Mendelian randomization analysis

Mendelian randomization (MR) analysis that leverages genetic information is a statistical analog of randomized controlled trials [17]. Since genotypes were allocated during meiosis and were independent of environmental exposures, MR estimates were deemed to be free of confounders and widely used for causal inference in epidemiological studies [17]. Traditional MR analysis often presumes a linear relationship between exposure and outcome. Herein, to examine the potential nonlinear relationship between alcohol intake and HCC, we conducted a nonlinear MR (NLMR) analysis (Additional file 3: Method S2) [18]. Six SNPs (rs11940694, rs1229984, rs1302808, rs55872084, rs7187575, and rs676388) were used in the NLMR analysis [19]. Odds ratio (OR) and its 95% CI were calculated to quantify the association between genetically determined alcohol intake and HCC risk. Two tests for nonlinearity are reported: a trend test, which assesses a linear trend among the localized average causal effect estimates, and a fractional polynomial test, which assesses whether a nonlinear model fits the localized average causal effect estimates better than a linear model [20]. All statistical analyses were performed using R program (version 4.0.3). *P* value < 0.05 was deemed as statistically significant.

## Results

### Characteristics of study participants

Of 329,164 participants (52.3% females, mean [standard deviation] age = 56.7 [8.0] years), 201 incident HCC cases (23.4% females, mean age = 62.3 [5.6] years, incidence rate = 4.83/100,000) occurred during the median follow-up of 12.6 years (interquartile: 11.9–13.3 years) (Table 1). The HCC incidence was significantly lower in women, people aged < 60 years, subjects with normal ALT levels, and those carrying non-risk genotypes of *PNPLA3* rs738409 and *TM6SF2* rs58542926 than in their respective counterparts. Individuals who had low-to-moderate drinking and those who drank less frequently showed a lower HCC incidence than those who abstained (Table 1). There were 12,342; 190,180; 89,012; and 37,630 individuals identified as nondrinkers, low-to-moderate, excess, and heavy drinkers, respectively (Additional file 2: Table S3). The baseline characteristics were distinct among subgroups by alcohol intake level (Additional file 2: Table S3). For example, compared with nondrinkers, the proportions of males, former and current smokers, individuals with higher income and those overweight (BMI ≥ 25), and frequency of the rs738409 CC genotype were increased with alcohol intake. Low-to-moderate drinkers consumed more wine than the other three groups. HBV/HCV seropositivity was comparable among the four groups (Additional file 2: Table S3).

### Association of pure alcohol intake level with HCC risk

**Fig. 1:**
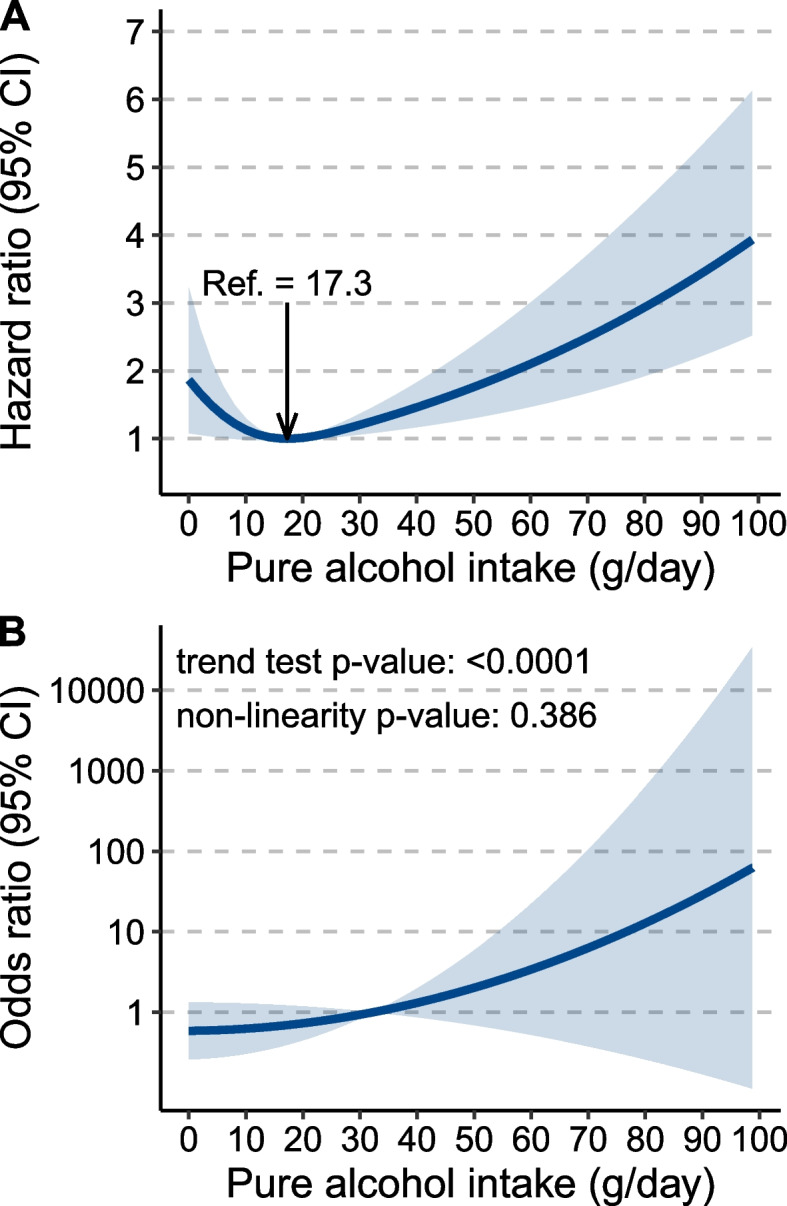
Association of alcohol consumption with the risk of hepatocellular carcinoma (HCC). **A** J-shaped correlation pattern derived from the additive Cox regression model, in which the degree of freedom of alcohol use was 3. **B** Linear relationship derived from nonlinear Mendelian randomization analysis

The AIC values of additive Cox regression models with different *df* in overall subjects and in subgroups are shown in Additional file 2: Table S4. The AIC-minimized model (*df* = 3) suggested a J-shaped relationship between the daily pure alcohol intake level and HCC risk (Fig. 1A). A similar J-shaped correlation pattern was also observed in subgroups by alcohol intake frequency and whether alcohol was consumed with a meal (Additional file 1: Figs. S2-3). However, NLMR analysis yielded a *P-*value of 0.386 for the fractional polynomial test and a *P*-value < 0.0001 for the trend test, suggesting a linear relationship between genetically determined alcohol intake level and HCC risk (Fig. 1B; Additional file 2: Table S5).

**Fig. 2:**
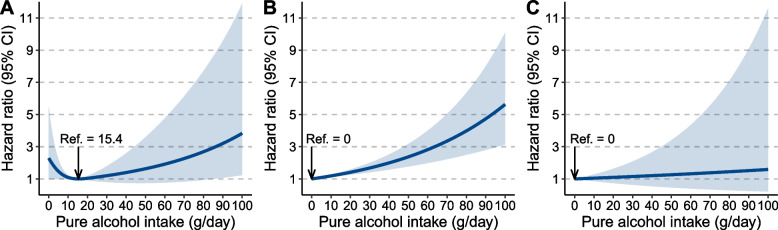
Association of alcohol consumption with the risk of hepatocellular carcinoma (HCC) by predominant alcohol beverage type. **A** Wine; **B** beer; and **C** spirits and fortified wine. The degrees of freedom of alcohol use were 3, 1, and 1, respectively

We observed a J-shaped correlation between alcohol intake and HCC risk in individuals who mainly drank wine (Fig. 2A). However, in participants who mainly drank beer and spirits and fortified wine, we found that the risk of HCC was proportionally increased with the amount of alcohol intake (Fig. 2B, C). The HR was not statistically significant across the volume of alcohol intake in subpopulations predominantly drank spirits and fortified wine, which might be largely ascribed to the small case number of HCC in this population.

### Association of pure alcohol intake level with HCC risk in subgroups by sex, age, ALT levels, and genetic variants

The correlations between alcohol intake level and HCC risk varied by sex, age, and baseline ALT levels (Additional file 1: Fig. S4). The J-shaped correlation pattern was detected in women, people aged < 60 years, and those with a normal ALT level (< 40 U/L). However, the associations of alcohol intake level with HCC risk were shown in a positive dose–response manner in men, people aged ≥ 60 years, and those with an abnormal ALT level.

The association of alcohol consumption with HCC risk was modified by genetic variants (Additional file 1: Fig. S5). In participants carrying non-risk genotypes of *PNPLA3* rs738409 and *TM6SF2* rs58542926, we observed a J-shaped correlation between alcohol use and HCC risk. However, in individuals carrying the risk alleles, the risk of HCC proportionally increased with the level of alcohol intake.

### The effect of drinking wine on HCC risk in subgroups

**Fig. 3:**
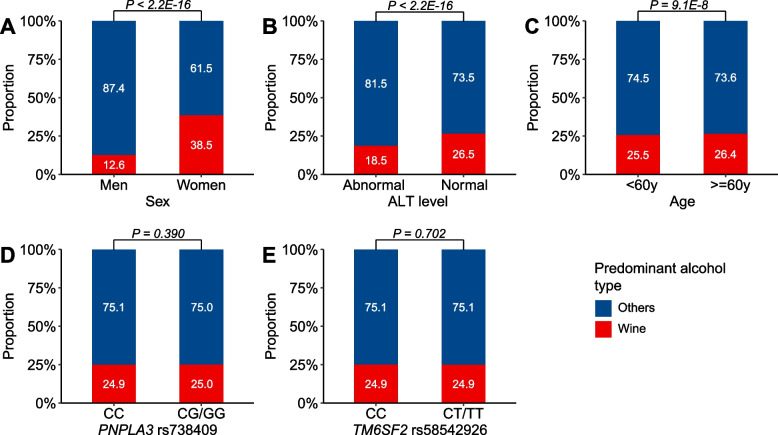
The proportions of subjects who mainly drank wine in subgroups by sex, baseline alanine transaminase (ALT) levels, age, and genotypes of *PNPLA3* rs738409 and *TM6SF2* rs58542926. The *P* values were calculated from the chi-squared test for counts in subgroups

Women and participants with normal ALT levels drank more wine than their respective counterparts (Fig. 3A, B). Of note, we observed a linear relationship between alcohol use and HCC risk in women and in individuals who had normal ALT levels when excluding those who mainly drank wine (Additional file 1: Figs. S6 and S7).

There were 26.4% of the elders majorly drinking wine, which was significantly higher than the younger population (Fig. 3C). The proportion of participants predominantly drinking wine was comparable between genotypes of both *PNPLA3* rs738409 and *TM6SF2* rs58542926 (Fig. 3D, E). Likewise, we observed a positive dose–response relationship between alcohol consumption and HCC risk in people aged < 60 years and in those carrying the CC genotype of *TM6SF2* rs58542926 after the exclusion of subjects mainly intaking wine (Additional file 1: Figs. S8 and S9). However, the J-shaped correlation remained in people carrying the CC genotype of *PNPLA3* rs738409, even excluding those mainly drinking wine (Additional file 1: Fig. S10). The AIC values for the additive Cox regression models in the subgroup analyses are shown in Additional file 2: Table S6.

To test whether wine drinking confers a beneficial effect on HCC development in men, older people, subjects with abnormal ALT levels, and individuals carrying risk alleles of *PNPLA3* rs738409 and *TM6SF2* rs58542926, we performed additive Cox regression models in the subpopulations who mainly drank wine. The AIC-minimized models suggested a linear relationship between alcohol intake and HCC risk in all subgroups, although the HR estimates were statistically nonsignificant in men, subjects with abnormal ALT levels, and those carrying the CT/TT genotypes of *TM6SF2* rs58542926 (Additional file 1: Figs. S11-S15 and Additional file 2: Table S7).

### Moderate wine drinking decreases serum levels of ALT and AST

**Fig. 4:**
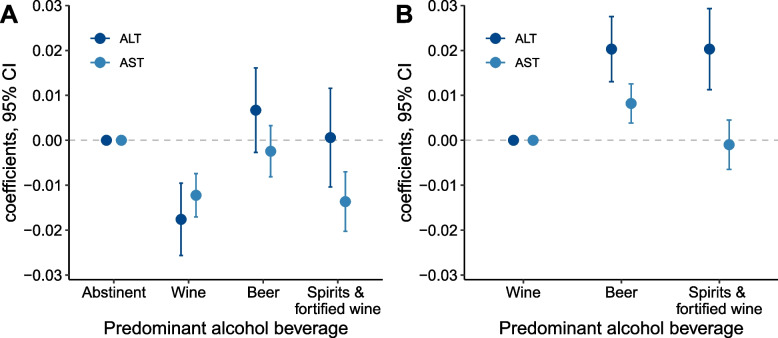
The associations of low-to-moderate drinking with serum levels of alanine transaminase (ALT) and aspartate aminotransferase (AST). **A** ALT and AST levels were log-transformed. Age and sex were adjusted in linear regression models with the abstinent set as the reference group. **B** ALT and AST levels were log-transformed. Age, sex, and alcohol intake amount were adjusted in the linear regression models with the wine set as the reference group

The serum levels of ALT and AST increased with increasing alcohol intake (Additional file 2: Table S3). We performed a linear regression model that adjusted for age and sex to assess the effect of moderate drinking on serum levels of ALT and AST. Compared with the abstinent, moderate wine drinking significantly decreased the circulating levels of both ALT and AST (Fig. 4A). When further adjusting for the amount of alcohol intake, we found that moderate drinking of beer and spirits and fortified wine significantly increased the ALT level in contrast to moderate wine drinking (Fig. 4B). The ALT- and AST-lowering effect of moderate wine drinking was more pronounced in women, subjects aged < 60 years, and those carrying the non-risk genotype of *TM6SF2* rs58542926 than in their respective counterparts (Additional file 1: Figs. S16-S18), whereas it was comparable in those carrying the CC and CG/GG genotypes of *PNPLA3* rs738409 (Additional file 1: Fig. S19).

## Discussion

In this prospective cohort study, we reported a J-shaped correlation between the daily pure alcohol intake level and the risk of HCC, indicating that low-to-moderate drinking may be inversely associated with HCC risk. The J-shaped correlation pattern was detected only in subjects who mainly drank wine but not in those who mainly drank beer and spirits and fortified wine, suggesting that the beneficial effect of low-to-moderate drinking may be largely attributed to wine drinking. We also noted that the association between alcohol consumption and HCC varied by sex, age, ALT level, and genetic variants. The observed J-shaped correlation was more marked in the low-risk populations of HCC.

Alcohol consumption (or ethanol exposure) is one of the well-determined risk factors for HCC, and the underlying mechanisms have been extensively investigated [21, 22]. Although heavy drinking is well associated with the risk of HCC, whether low-to-moderate drinking inversely associated with HCC risk is still under debate. In this case, cohort studies with large sample sizes, long follow-up times, and particularly detailed information on alcohol exposure are warranted. Our study involved nearly 0.33 million subjects and found a J-shaped association between alcohol use and HCC risk, confirming a previous finding that low-to-moderate drinking is inversely associated with HCC risk [8]. The Liver Cancer Pooling Project involving more than 1.5 million participants revealed that light-to-moderate consumption of any type of alcohol was associated with a decreased HCC risk [8]. However, in this study, most participants were assessed for alcohol use over the past year rather than lifetime exposure, which may lead to the misclassification of previous heavy drinkers as abstinent. Additionally, the effect of specific types of alcohol on HCC might be entangled by other alcohol because the predominant alcohol type was not assessed for each participant. The Million Women Study involved approximately 1.3 million women and reported a 41% increased risk of liver cancer in the nondrinkers compared to those drinking ≤ 2 drinks per week [23]. However, the lifelong nondrinkers were not teased out from those who had stopped drinking. Compared to the two largest cohort studies, our study yielded a similar finding and provided a more detailed dissection of the association between alcohol use and HCC. A recent umbrella review of meta-analyses also showed that low alcohol consumption was associated with a 27% (95% CI 2–46%) decreased risk of liver cancer [9]. In contrast, some studies reported no association or a proportionally increasing correlation between alcohol use and the risk of liver cancer [10, 24–26]. The between-study inconsistencies may attributed to many reasons, including study design (e.g., case–control study vs. cohort study), sample size and follow-up time, genetic background and HCC incidence of study participants, and more importantly, the recall bias due to documentation and calculation of lifetime alcohol use.

In the present study, we found that the putative protective effect of low-to-moderate drinking on HCC may be largely attributed to wine intake. Indeed, moderate wine drinking has been reported to reduce the risk of chronic diseases including liver fibrosis [27], cirrhosis [28–30], venous thromboembolism [31], and esophageal disorders [32]. In addition, we found that low-to-moderate wine drinking decreases the serum levels of ALT and AST, the leading biomarkers for liver injury in clinical practice, compared with the nondrinkers. Previous studies suggested that polyphenols, the bioactive components in wine including anthocyanin, resveratrol, and gallic acid, may have antioxidant properties, including regulating lipid metabolism and microbiota composition, attenuating ethanol-induced oxidative stress, and anticarcinogenesis [33–35]. Moreover, moderate wine drinkers appear to be at lower risk of becoming heavy drinkers and subjects who preferred wine had healthier diets than those who preferred beer or spirits [36–38], which may further explain the “positive role” of wine drinking.

The association of alcohol use with HCC significantly varied by sex, age, ALT level, and genetic variants. The HCC incidence rates were significantly lower in women, people aged < 60 years, subjects with normal ALT levels, and those carrying non-risk genotypes of *PNPLA3* rs738409 and *TM6SF2* rs58542926 than in their respective counterparts, although the age of HCC diagnoses was comparable. We observed a J-shaped relationship between alcohol consumption and HCC risk in the HCC low-risk populations. However, when excluding participants mainly drinking wine, alcohol use was associated with HCC risk in a positive dose–response manner in all subpopulations except those with the CC genotype of *PNPLA3* rs738409. The results further validate the protective effect of moderate wine drinking on hepatic carcinogenesis. Of note, in the high-risk subgroups of HCC, including men, people aged ≥ 60 years, subjects with abnormal ALT levels, and those carrying the risk alleles of *PNPLA3* rs738409 and *TM6SF2* rs58542926, we observed a linear relationship between alcohol use and HCC risk, even in those mainly drinking wine. Therefore, drinking alcohol should be completely abstained from in these subpopulations.

Although the current evidence implies that the inverse association between low-to-moderate drinking and HCC may be shaped by bioactive components in wine, the health impact of alcohol (or ethanol) also warrants more investigation. Our nonlinear MR analysis suggested a linear correlation between alcohol intake and HCC, indicating that alcohol per se may have no beneficial effect on the liver. This finding was consistent with prior MR studies on the association of alcohol with cardiovascular diseases and NAFLD [39–41]. However, in our previous study, we found that low-to-moderate drinking regardless of alcohol type was inversely associated with liver fat content [12], which was also observed in mouse models [42, 43]. In the current study, the J-shaped correlation between alcohol use and HCC in participants carrying the CC genotype of *PNPLA3* rs738409 was consistent among alcohol types. While the findings seem to indicate a potentially positive effect of alcohol [44], we should bear in mind that the beneficial effect may only show in a proportion of people.

Our study has some strengths. In contrast to previous studies that were mainly limited to subjects with fatty liver diseases or cirrhosis [45–47], we included relatively healthy people to obviate the bias from alcohol drinking behavior. Second, the effect of alcohol intake was assessed in a continuous form in our study instead of a conventional categorical form as in previous studies [7, 8, 48], thus preventing the loss of data information and enabling more precise estimates based on the additive Cox regression model. Third, the effect of a specific alcohol beverage type on HCC was evaluated with the exclusion of other alcohols. The confounding effect of alcohol types was therefore largely negated.

Given the strengths, our study also has limitations. First, alcohol use was acquired from a self-report questionnaire, which might be subject to recall bias and underreporting in heavy drinkers. However, there are no ideal biomarkers to measure the lifetime exposure of alcohol. Elaborate questionnaires are an acceptable alternative and have been widely used in epidemiological studies [39, 49]. Second, the case number of HCC was small in our study, despite the large sample size. The small case number undermined the robustness of modeling estimates, representing by the wide confidence intervals in certain subgroups. In this case, our findings should be interpreted with cautions and need be further validated in cohort study with large case number. However, the association pattern between alcohol use and HCC risk remained consistent in the homogeneous subgroups (e.g., low-risk groups of HCC). Moreover, fitting the volume of alcohol consumption in a continuous form instead of a categorical form in the additive Cox regression model to some extent ameliorates the impact of a small case number [50]. Our findings therefore give novel clues to the association of alcohol consumption with HCC. Third, the mean age of HCC diagnosis in the UK Biobank participants was 62.3 years, which was younger than the average age of HCC diagnosis in the UK (71.2 years) [51], indicating that the current follow-up time is not sufficient. Thus, a longer follow-up time is warranted to identify more HCC cases, thereby increasing the statistical power. Fourth, HBV/HCV status was available for only a small proportion of study participants. However, for individuals with HBV/HCV status available, there was no association between these potential covariates and alcohol consumption and no overlap between HCC cases and participants who were positive for HBV/HCV, suggesting that HBV/HCV might have little impact on the association between alcohol use and HCC. Finally, our study participants were of European ancestry, thus limiting the extrapolation of our findings to other populations because of the differences in both alcohol drinking habits and genetic background.

## Conclusions

In summary, our findings may suggest a J-shaped correlation between alcohol consumption and HCC risk in low-risk populations of HCC, such as women, people aged < 60 years, and those with normal ALT levels and non-risk genetic variants. This correlation pattern may be largely driven by wine drinking. However, in high-risk populations, drinking any kind of alcohol should be abstained from to reduce the risk of HCC.

## Supplementary Information

**Additional file 1: Fig. S1.** Flow chart for selection of study participants. **Fig. S2.** Association between alcohol intake level and risk of HCC in subgroups by alcohol intake frequency. **Fig. S3.** Association between alcohol intake level and risk of HCC in subgroups by whether taken alcohol with meal. **Fig. S4.** Association of alcohol consumption with the risk of HCC by sex, age, and baseline ALT levels. **Fig. S5.** Association of alcohol consumption with the risk of HCC by genotypes of PNPLA3 rs738409 and TM6SF2 rs58542926. **Fig. S6.** Association between alcohol intake and risk of HCC in women, excluding those mainly drank wine. **Fig. S7.** Association between alcohol intake and risk of HCC in individuals with normal ALT levels, excluding those mainly drank wine. **Fig. S8.** Association between alcohol intake and risk of HCC in people aged < 60 years, excluding those mainly drank wine. **Fig. S9.** Association between alcohol intake and risk of HCC in individuals carrying CC genotype of TM6SF2 rs58542926, excluding those mainly drank wine. **Fig. S10.** Association between alcohol intake and risk of HCC in individuals carrying CC genotype of PNPLA3 rs738409, excluding those mainly drank wine. **Fig. S11.** Association between alcohol intake and risk of HCC in men mainly drinking wine. **Fig. S12.** Association between alcohol intake and risk of HCC in people aged > = 60 years and mainly drank wine. **Fig. S13.** Association between alcohol intake and risk of HCC in individuals had abnormal ALT level and mainly drank wine. **Fig. S14.** Association between alcohol intake and risk of HCC in individuals carrying CC/GG genotype of PNPLA3 rs738409 and mainly drank wine. **Fig. S15.** Association between alcohol intake and risk of HCC in individuals carrying CT/TT genotype of TM6SF2 rs58542926 and mainly drank wine. **Fig. S16.** Effects of moderate drinking on serum levels of ALT and AST by sex. **Fig. S17.** Effects of moderate drinking on serum levels of ALT and AST by age. **Fig. S18.** Effects of moderate drinking on serum levels of ALT and AST by genotypes of TM6SF2 rs58542926. **Fig. S19.** Effects of moderate drinking on serum levels of ALT and AST by genotypes of PNPLA3 rs738409.

**Additional file 2: Table S1.** Calculation of pure alcohol intake in the UK Biobank cohort. **Table S2.** Prevalent and incident diseases in the UK Biobank cohort. **Table S3.** Characteristics of the study participants by levels of alcohol drinking. **Table S4.** AIC values for the additive Cox regression models with different degree of freedom. **Table S5.** Statistics for the nonlinear Mendelian randomization analysis. **Table S6.** AIC values for the additive Cox regression models with the exclusion of people mainly drinking wine. **Table S7.** AIC values for the additive Cox regression models in people mainly drinking wine.

**Additional file 3: Method S1.** Additive Cox regression model. **Method S2.** Nonlinear Mendelian randomization analysis.

## Data Availability

All data were available in the UK Biobank, and subject to registration and application processes.

## Abbreviations

HCC: Hepatocellular carcinoma
NLMR: Nonlinear Mendelian randomization
ALT: Alanine transaminase
AST: Aspartate aminotransferase
