# Supplementary material for: Alcohol consumption and hepatocellular carcinoma: novel insights from a prospective cohort study and nonlinear Mendelian randomization analysis
Source: BMC Med. 2022 Oct 28;20:413. doi: 10.1186/s12916-022-02622-8 (PMC9615332; doi:10.1186/s12916-022-02622-8)
Supplement: Supplementary file 1 — Additional file 1: Fig. S1. Flow chart for selection of study participants. Fig. S2. Association between alcohol intake level and risk of HCC in subgroups by alcohol intake frequency. Fig. S3. Association between alcohol intake level and risk of HCC in subgroups by whether taken alcohol with meal. Fig. S4. Association of alcohol consumption with the risk of HCC by sex, age, and baseline ALT levels. Fig. S5. Association of alcohol consumption with the risk of HCC by genotypes of PNPLA3 rs738409 and TM6SF2 rs58542926. Fig. S6. Association between alcohol intake and risk of HCC in women, excluding those mainly drank wine. Fig. S7. Association between alcohol intake and risk of HCC in individuals with normal ALT levels, excluding those mainly drank wine. Fig. S8. Association between alcohol intake and risk of HCC in people aged < 60 years, excluding those mainly drank wine. Fig. S9. Association between alcohol intake and risk of HCC in individuals carrying CC genotype of TM6SF2 rs58542926, excluding those mainly drank wine. Fig. S10. Association between alcohol intake and risk of HCC in individuals carrying CC genotype of PNPLA3 rs738409, excluding those mainly drank wine. Fig. S11. Association between alcohol intake and risk of HCC in men mainly drinking wine. Fig. S12. Association between alcohol intake and risk of HCC in people aged > = 60 years and mainly drank wine. Fig. S13. Association between alcohol intake and risk of HCC in individuals had abnormal ALT level and mainly drank wine. Fig. S14. Association between alcohol intake and risk of HCC in individuals carrying CC/GG genotype of PNPLA3 rs738409 and mainly drank wine. Fig. S15. Association between alcohol intake and risk of HCC in individuals carrying CT/TT genotype of TM6SF2 rs58542926 and mainly drank wine. Fig. S16. Effects of moderate drinking on serum levels of ALT and AST by sex. Fig. S17. Effects of moderate drinking on serum levels of ALT and AST by age. Fig. S18. Effects of moderate drinking on seru [file 12916_2022_2622_MOESM1_ESM.docx]

Additional file 1: Figures S1-S19

Alcohol consumption and hepatocellular carcinoma: novel insights from a prospective cohort study and nonlinear Mendelian randomization analysis

Zhenqiu Liu, Ci Song, Chen Suo, Hong Fan, Tiejun Zhang, Li Jin, Xingdong Chen

TABLE OF CONTENT

[**Figure S1**. Flow chart for selection of study participants. 3](#_Toc116377814)

[**Figure S2**. Association between alcohol intake level and risk of hepatocellular carcinoma in subgroups by alcohol intake frequency. 4](#_Toc116377815)

[**Figure S3**. Association between alcohol intake level and risk of hepatocellular carcinoma in subgroups by whether taken alcohol with meal. 4](#_Toc116377816)

[**Figure S4**. Association of alcohol consumption with the risk of hepatocellular carcinoma (HCC) by sex, age, and baseline alanine transaminase (ALT) level. 5](#_Toc116377817)

[**Figure S5**. Association of alcohol consumption with the risk of hepatocellular carcinoma (HCC) by genotypes of PNPLA3 rs738409 and TM6SF2 rs58542926. 6](#_Toc116377818)

[**Figure S6**. Association between alcohol intake and risk of hepatocellular carcinoma in women, excluding those mainly drank wine. 7](#_Toc116377819)

[**Figure S7**. Association between alcohol intake and risk of hepatocellular carcinoma in individuals with normal ALT levels, excluding those mainly drank wine. 7](#_Toc116377820)

[**Figure S8**. Association between alcohol intake and risk of hepatocellular carcinoma in people aged < 60 years, excluding those mainly drank wine. 8](#_Toc116377821)

[**Figure S9**. Association between alcohol intake and risk of hepatocellular carcinoma in individuals carrying CC genotype of TM6SF2 rs58542926, excluding those mainly drank wine. 8](#_Toc116377822)

[**Figure S10**. Association between alcohol intake and risk of hepatocellular carcinoma in individuals carrying CC genotype of PNPLA3 rs738409, excluding those mainly drank wine. 9](#_Toc116377823)

[**Figure S11**. Association between alcohol intake and risk of hepatocellular carcinoma in men mainly drinking wine. 9](#_Toc116377824)

[**Figure S12**. Association between alcohol intake and risk of hepatocellular carcinoma in people aged >= 60 years and mainly drank wine. 10](#_Toc116377825)

[**Figure S13**. Association between alcohol intake and risk of hepatocellular carcinoma in individuals had abnormal ALT level and mainly drank wine. 10](#_Toc116377826)

[**Figure S14**. Association between alcohol intake and risk of hepatocellular carcinoma in individuals carrying CC/GG genotype of PNPLA3 rs738409 and mainly drank wine. 11](#_Toc116377827)

[**Figure S15**. Association between alcohol intake and risk of hepatocellular carcinoma in individuals carrying CT/TT genotype of TM6SF2 rs58542926 and mainly drank wine. 11](#_Toc116377828)

[**Figure S16**. Effects of moderate drinking on serum levels of ALT and AST by sex. 12](#_Toc116377829)

[**Figure S17**. Effects of moderate drinking on serum levels of ALT and AST by age. 12](#_Toc116377830)

[**Figure S18**. Effects of moderate drinking on serum levels of ALT and AST by genotypes of TM6SF2 rs58542926. 13](#_Toc116377831)

[**Figure S19**. Effects of moderate drinking on serum levels of ALT and AST by genotypes of PNPLA3 rs738409. 13](#_Toc116377832)

**Figure S1**. Flow chart for selection of study participants.


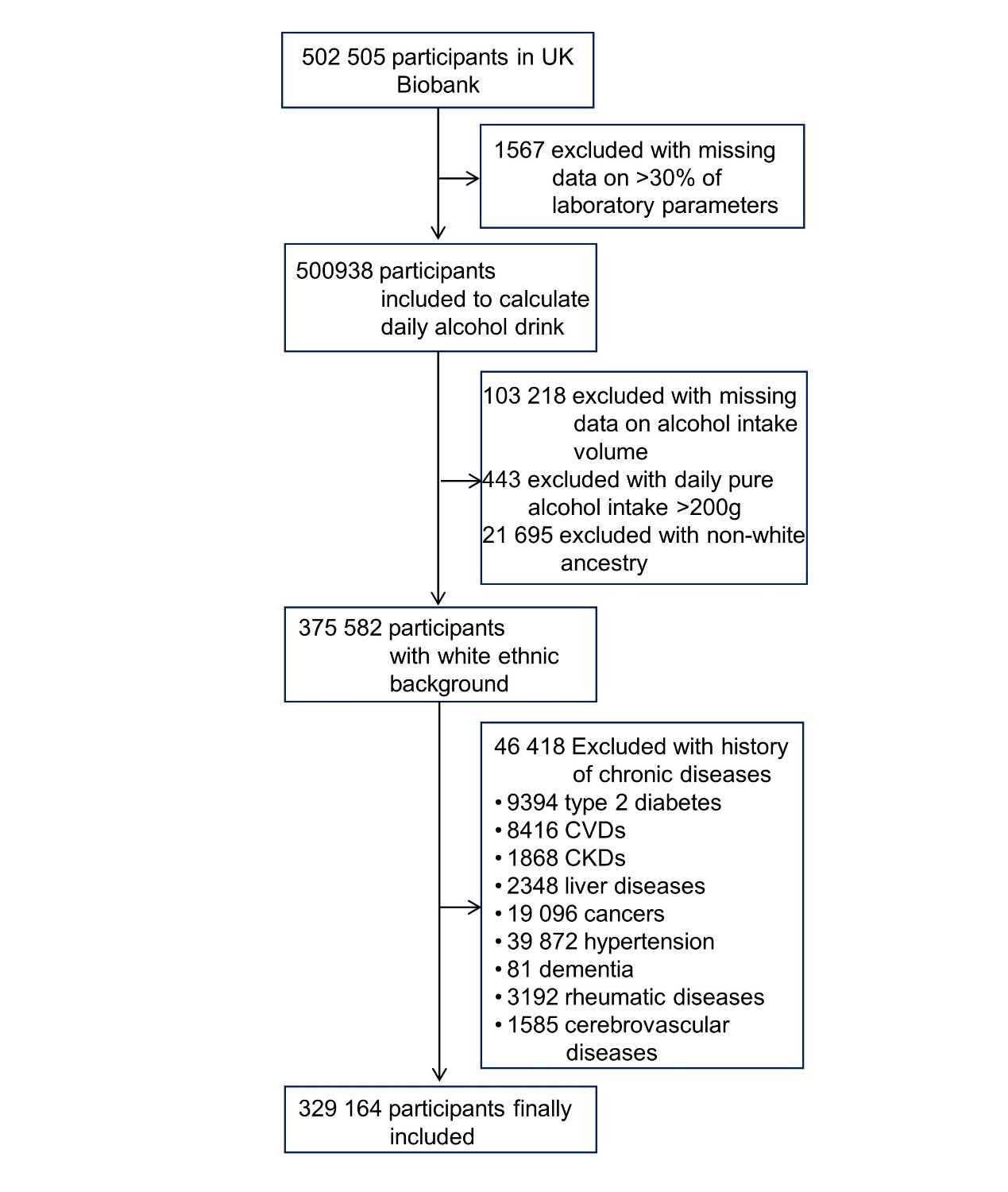


CVD, cardiovascular disease; CKD, chronic kidney disease.

**Figure S2**. Association between alcohol intake level and risk of hepatocellular carcinoma in subgroups by alcohol intake frequency.


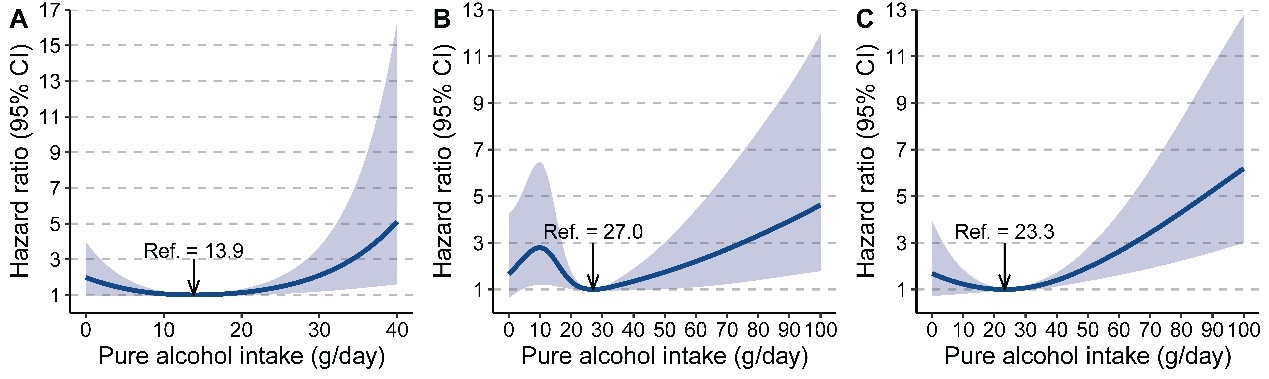


A, less-frequent; B, frequent; C, regular.

In less-frequent drinking group, the daily pure alcohol intake level was right-truncated to obviate the impact of outliers. Indeed, 97% (65/67) of total HCC cases were occurred in subjects had a pure alcohol intake < 40 g/day in this subgroup.

**Figure S3**. Association between alcohol intake level and risk of hepatocellular carcinoma in subgroups by whether taken alcohol with meal.


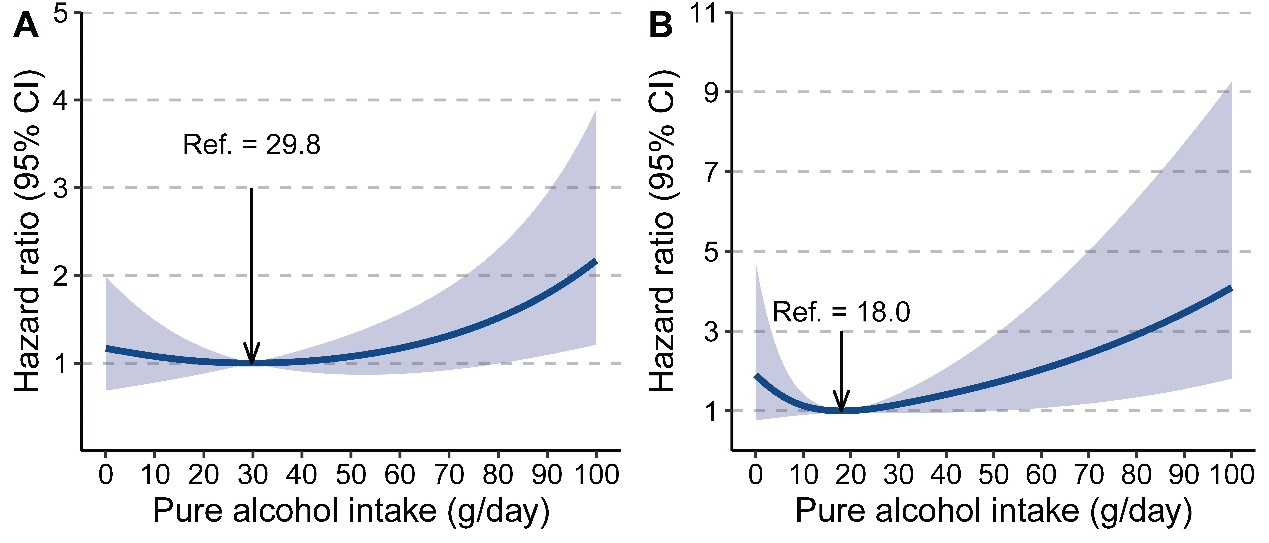


A, taken alcohol with meal; B, taken alcohol without meal.

**Figure S4**. Association of alcohol consumption with the risk of hepatocellular carcinoma (HCC) by sex, age, and baseline alanine transaminase (ALT) level.


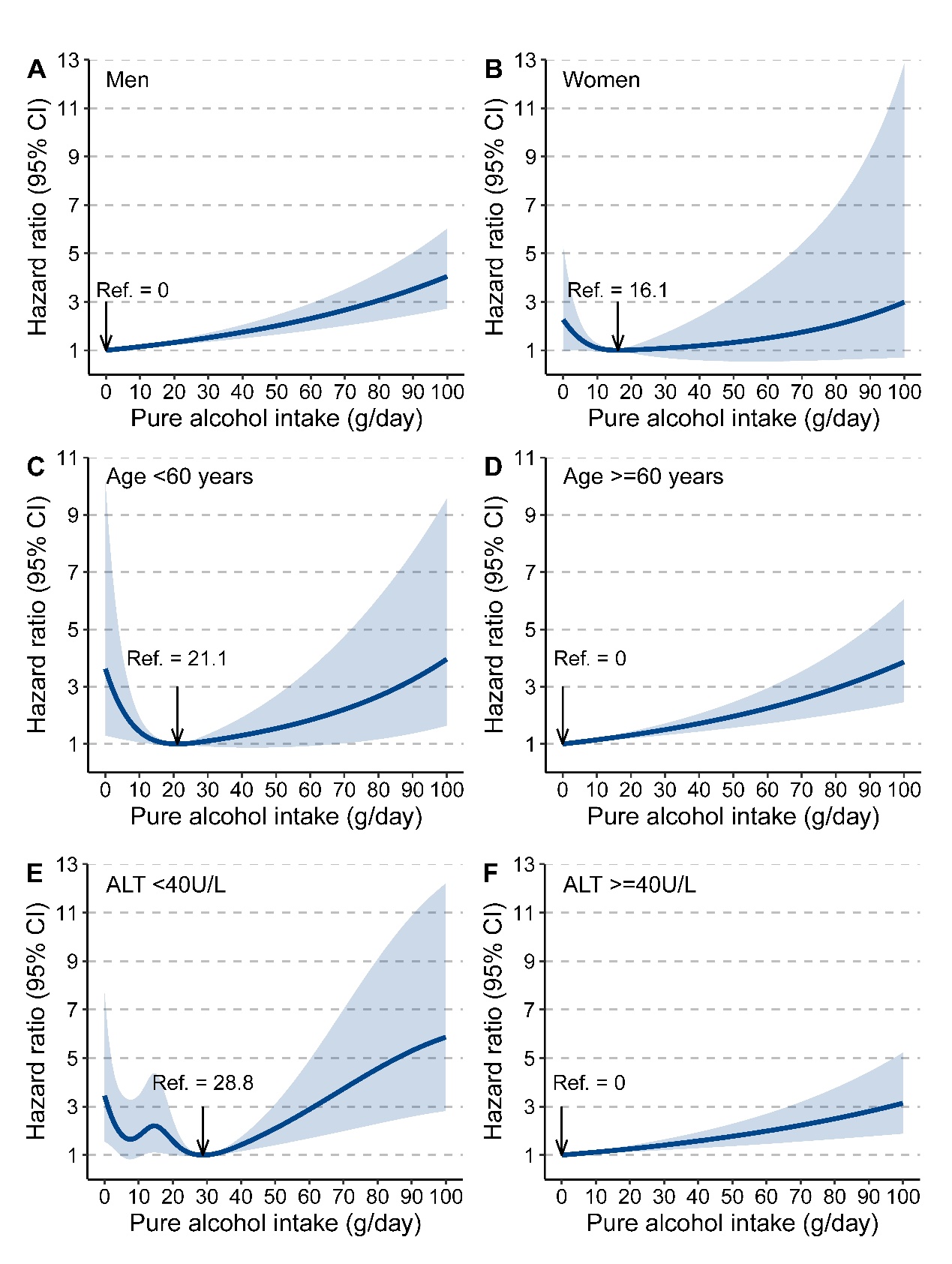


The degree of freedom of alcohol use was 1, 3, 3, 1, 5, and 1, respectively.

**Figure S5**. Association of alcohol consumption with the risk of hepatocellular carcinoma (HCC) by genotypes of PNPLA3 rs738409 and TM6SF2 rs58542926.


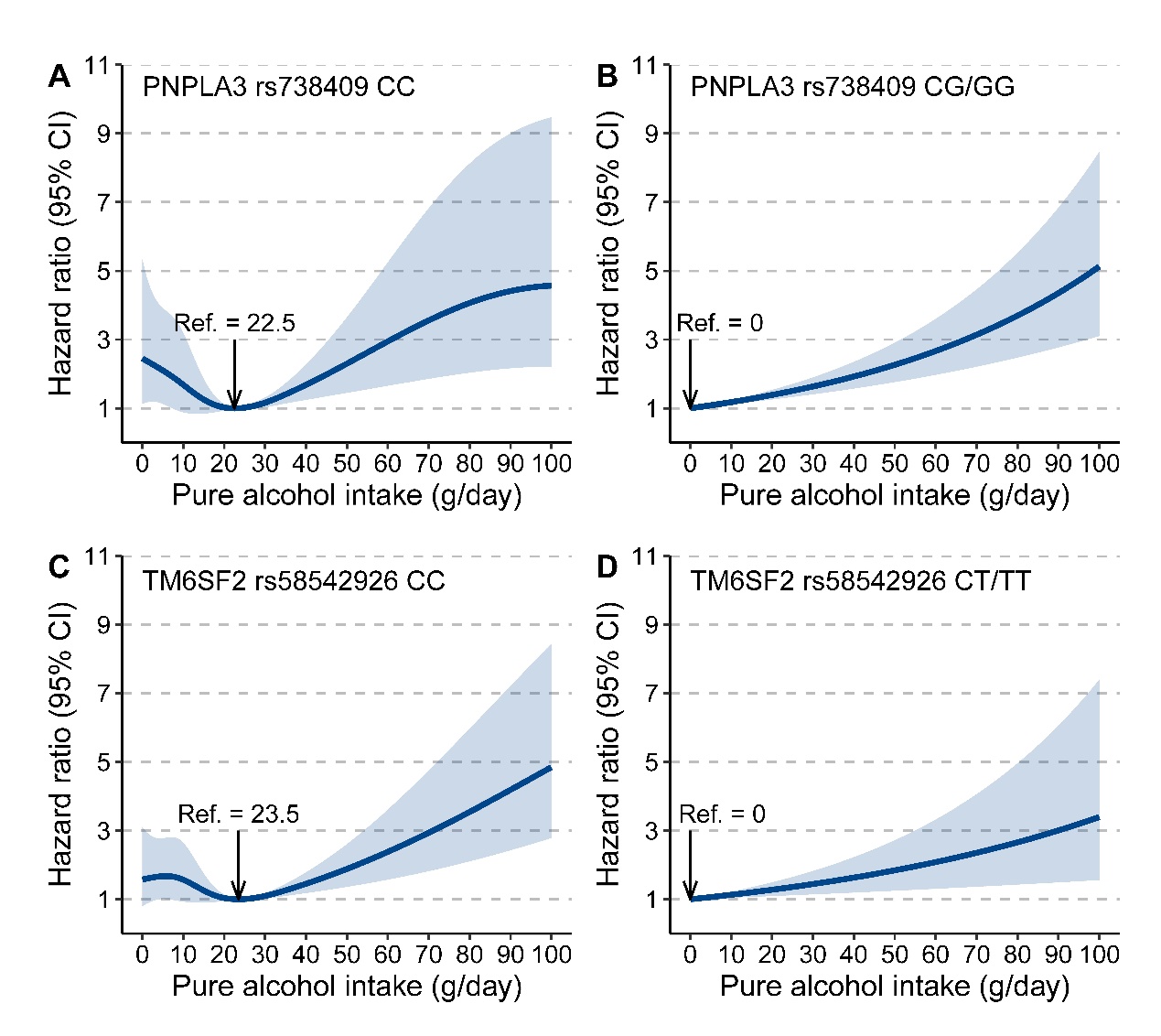


The degree of freedom of alcohol use was 4, 1, 4, and 1, respectively.

**Figure S6**. Association between alcohol intake and risk of hepatocellular carcinoma in women, excluding those mainly drank wine.


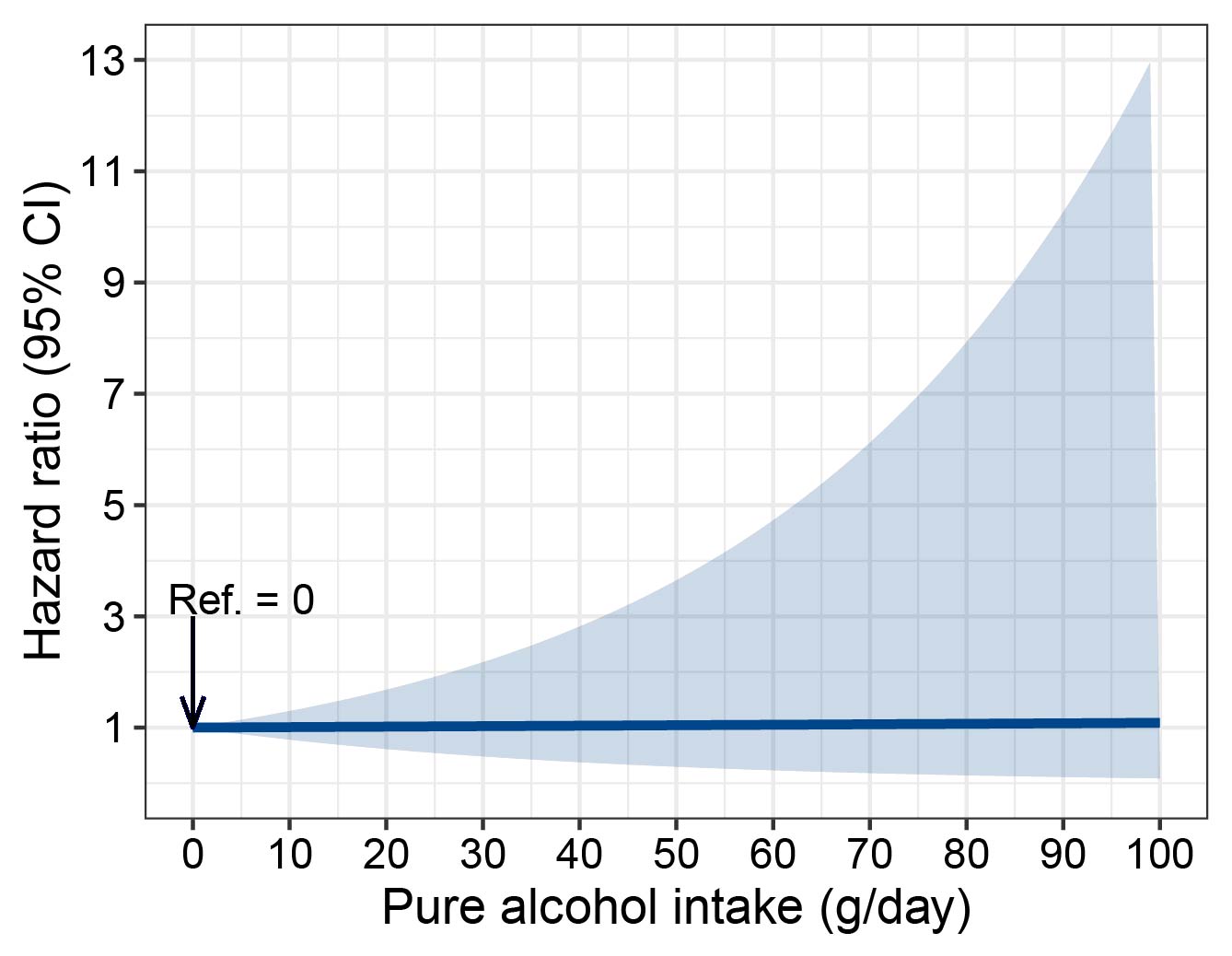


**Figure S7**. Association between alcohol intake and risk of hepatocellular carcinoma in individuals with normal ALT levels, excluding those mainly drank wine.


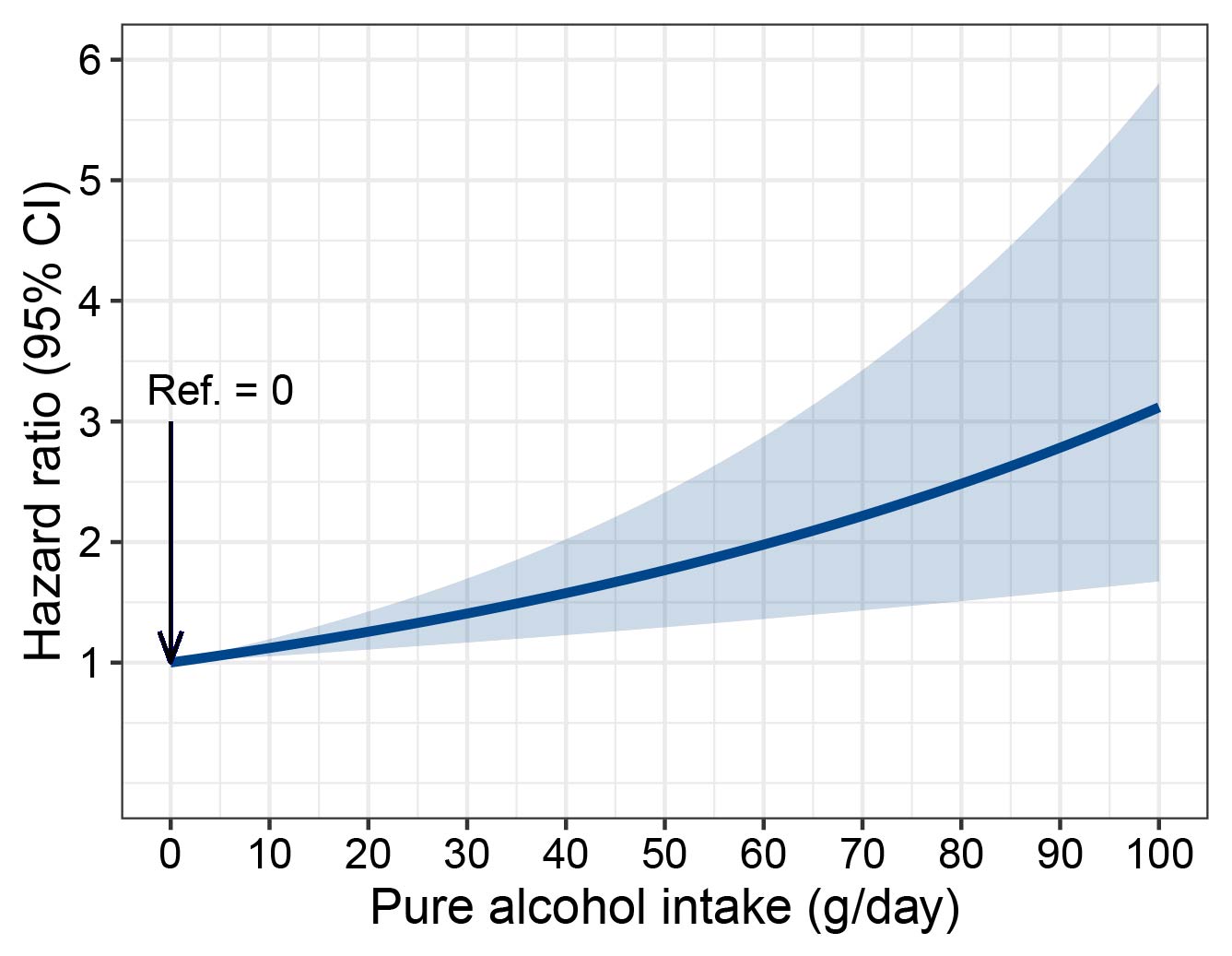


**Figure S8**. Association between alcohol intake and risk of hepatocellular carcinoma in people aged < 60 years, excluding those mainly drank wine.


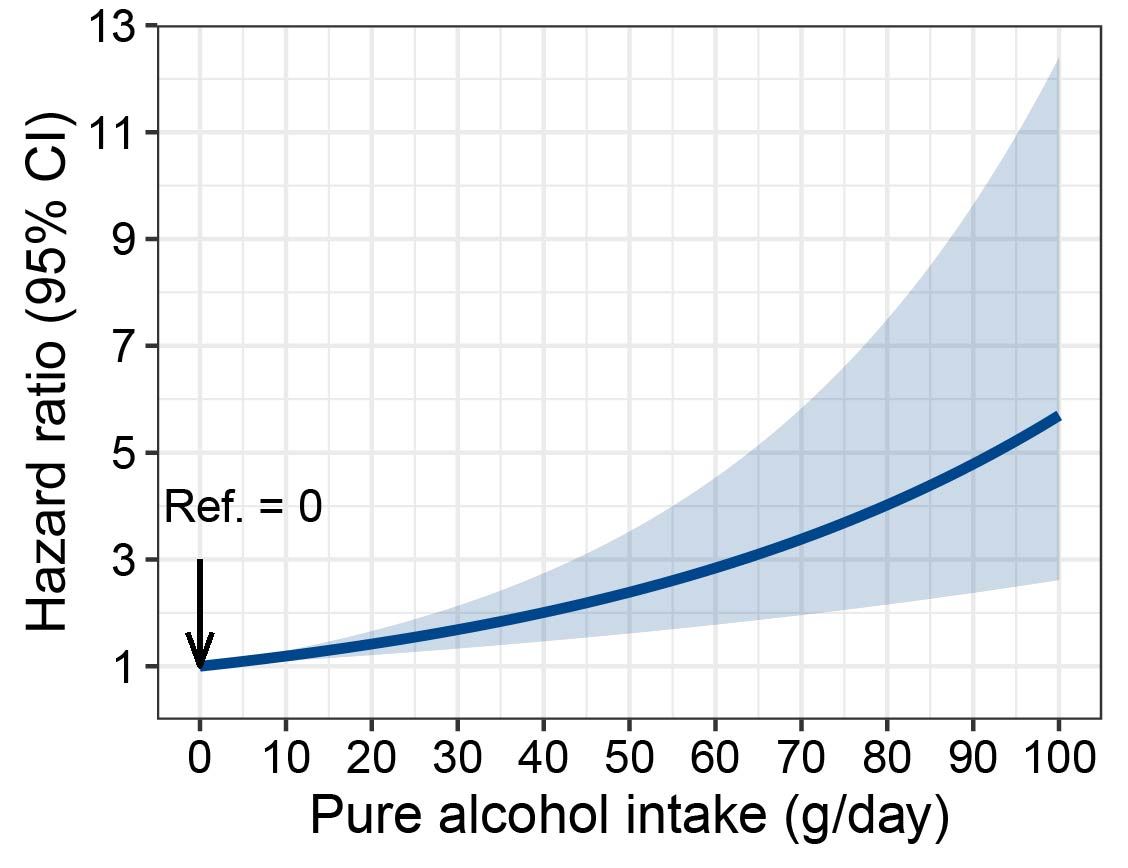


**Figure S9**. Association between alcohol intake and risk of hepatocellular carcinoma in individuals carrying CC genotype of TM6SF2 rs58542926, excluding those mainly drank wine.


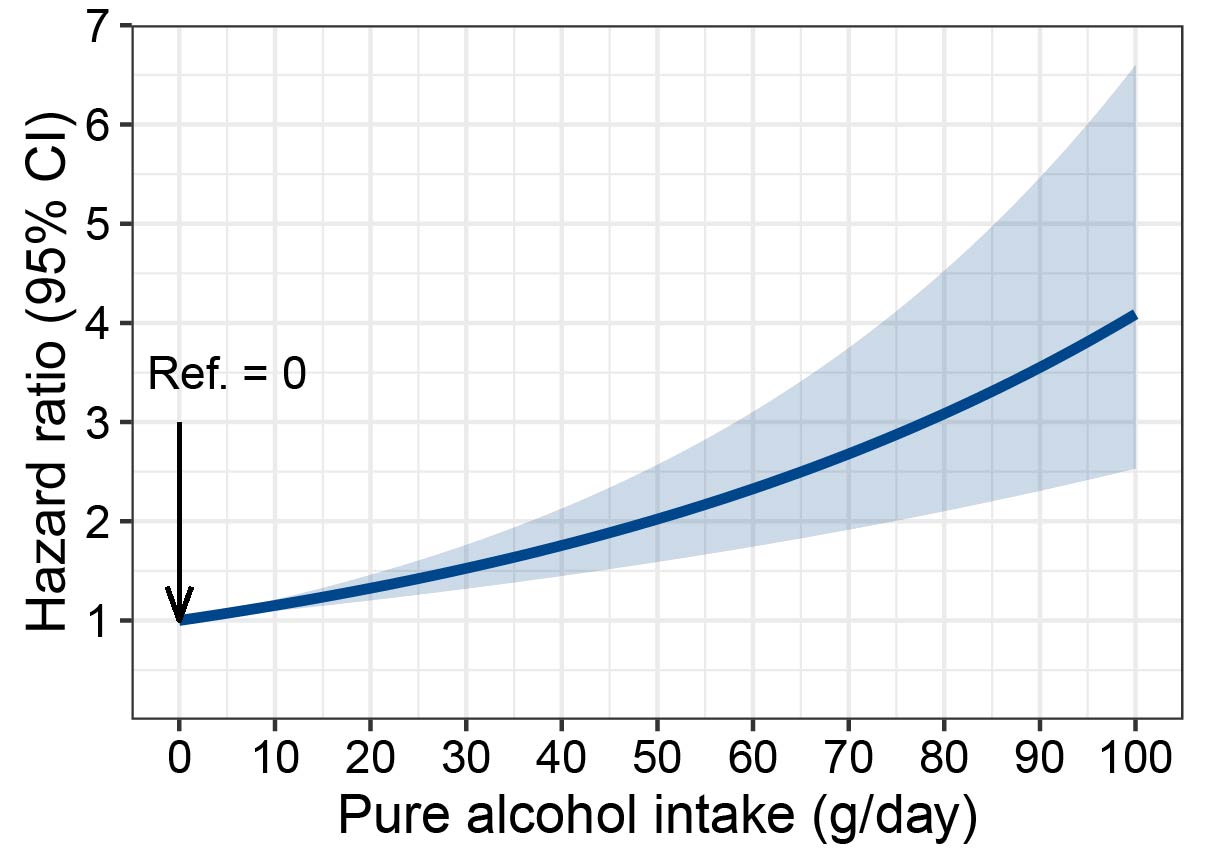


**Figure S10**. Association between alcohol intake and risk of hepatocellular carcinoma in individuals carrying CC genotype of PNPLA3 rs738409, excluding those mainly drank wine.


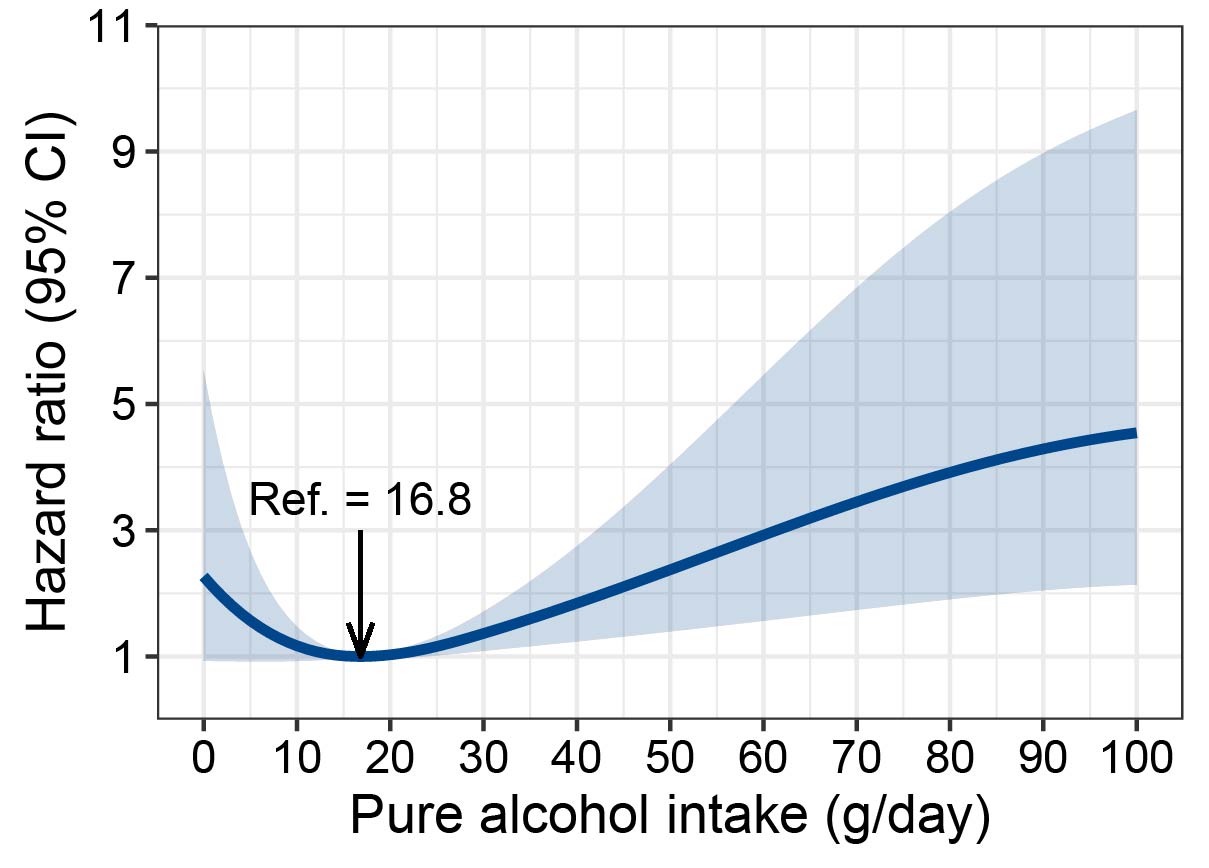


**Figure S11**. Association between alcohol intake and risk of hepatocellular carcinoma in men mainly drinking wine.


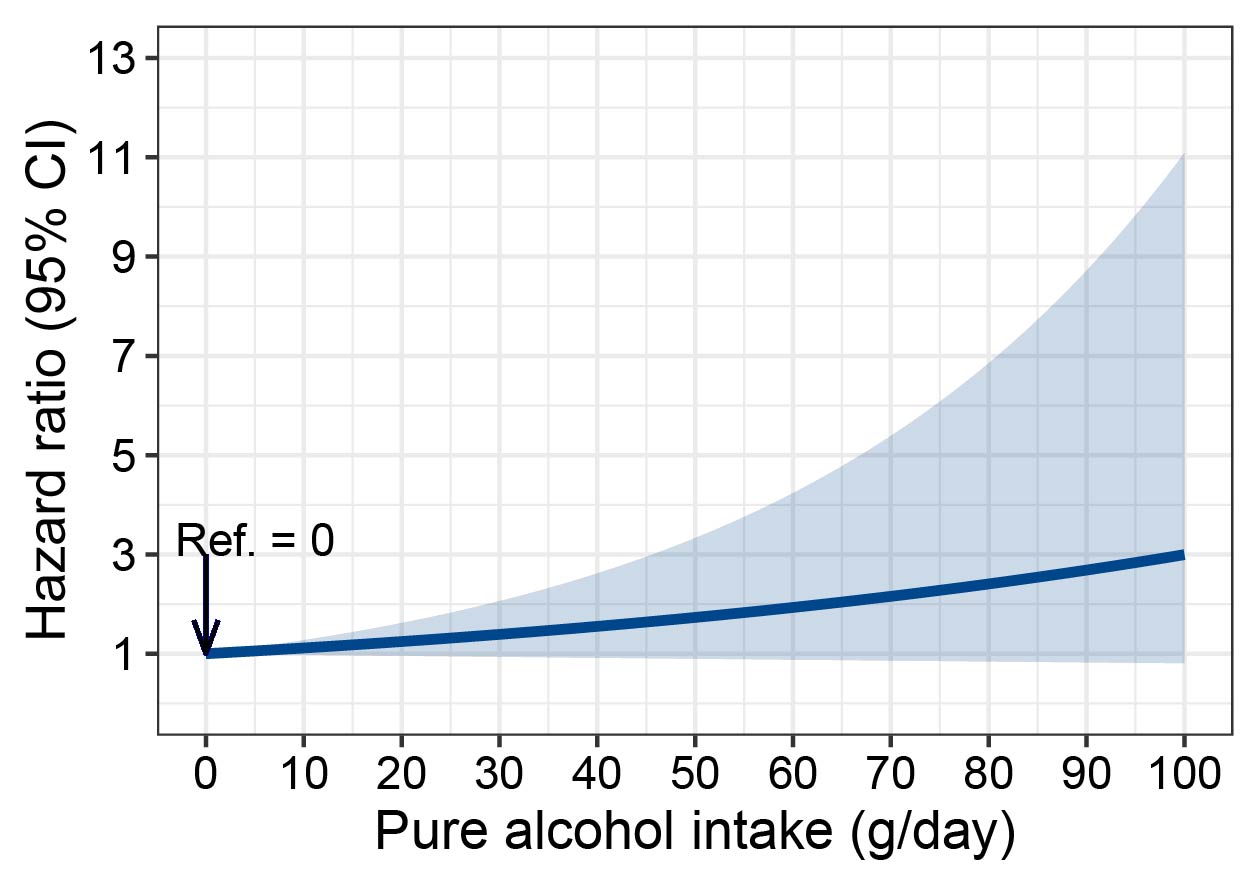


**Figure S12**. Association between alcohol intake and risk of hepatocellular carcinoma in people aged >= 60 years and mainly drank wine.


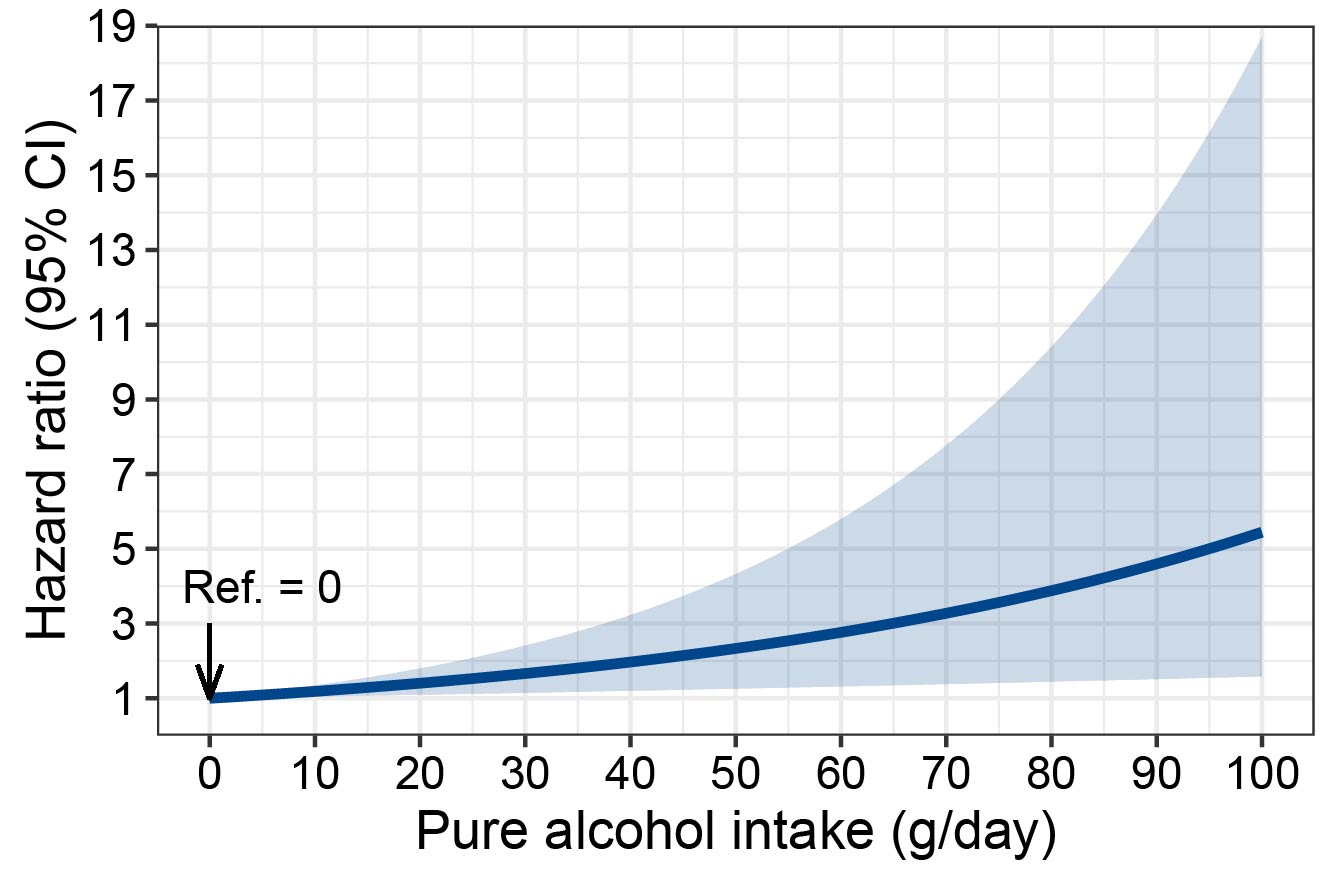


**Figure S13**. Association between alcohol intake and risk of hepatocellular carcinoma in individuals had abnormal ALT level and mainly drank wine.


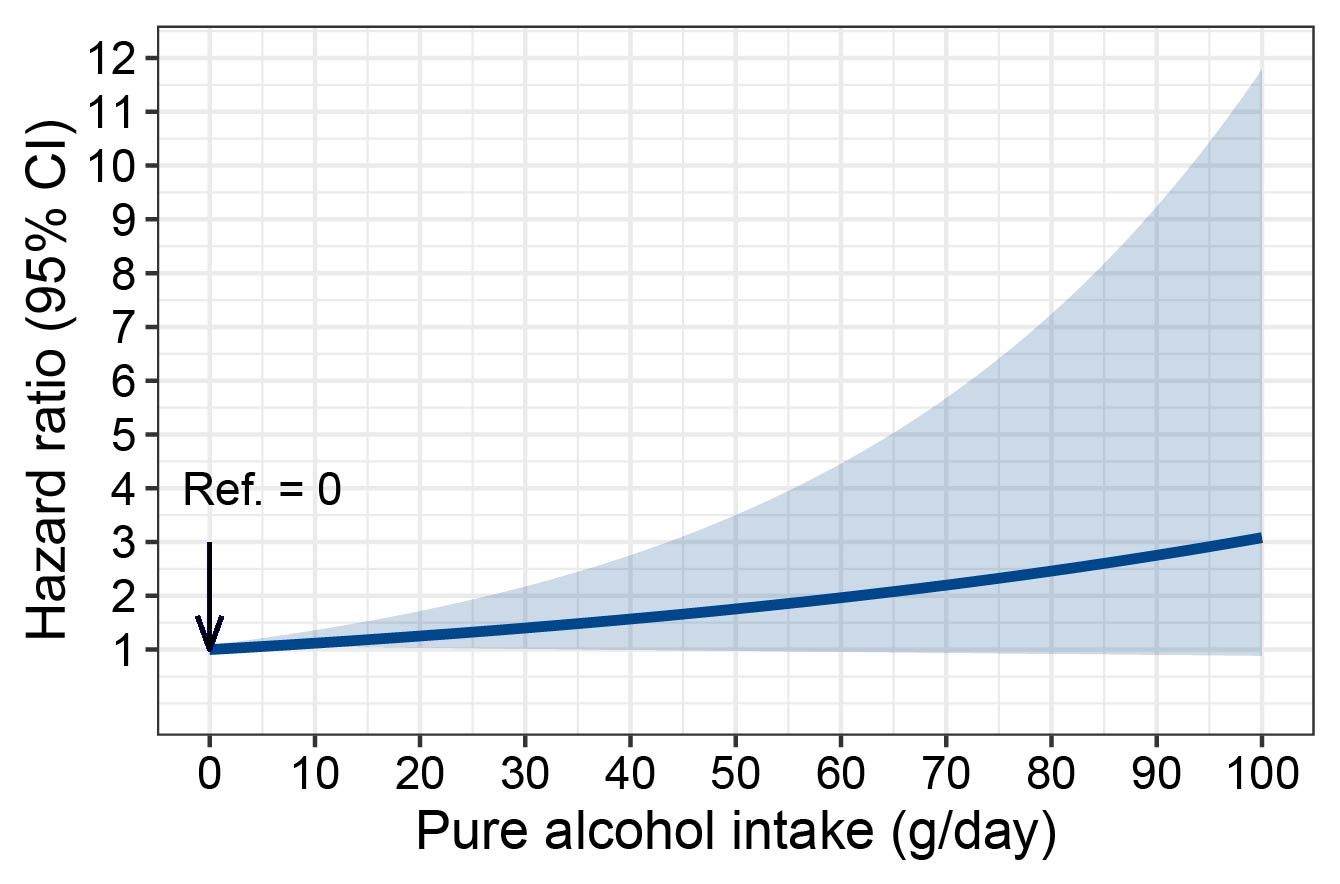


**Figure S14**. Association between alcohol intake and risk of hepatocellular carcinoma in individuals carrying CC/GG genotype of PNPLA3 rs738409 and mainly drank wine.


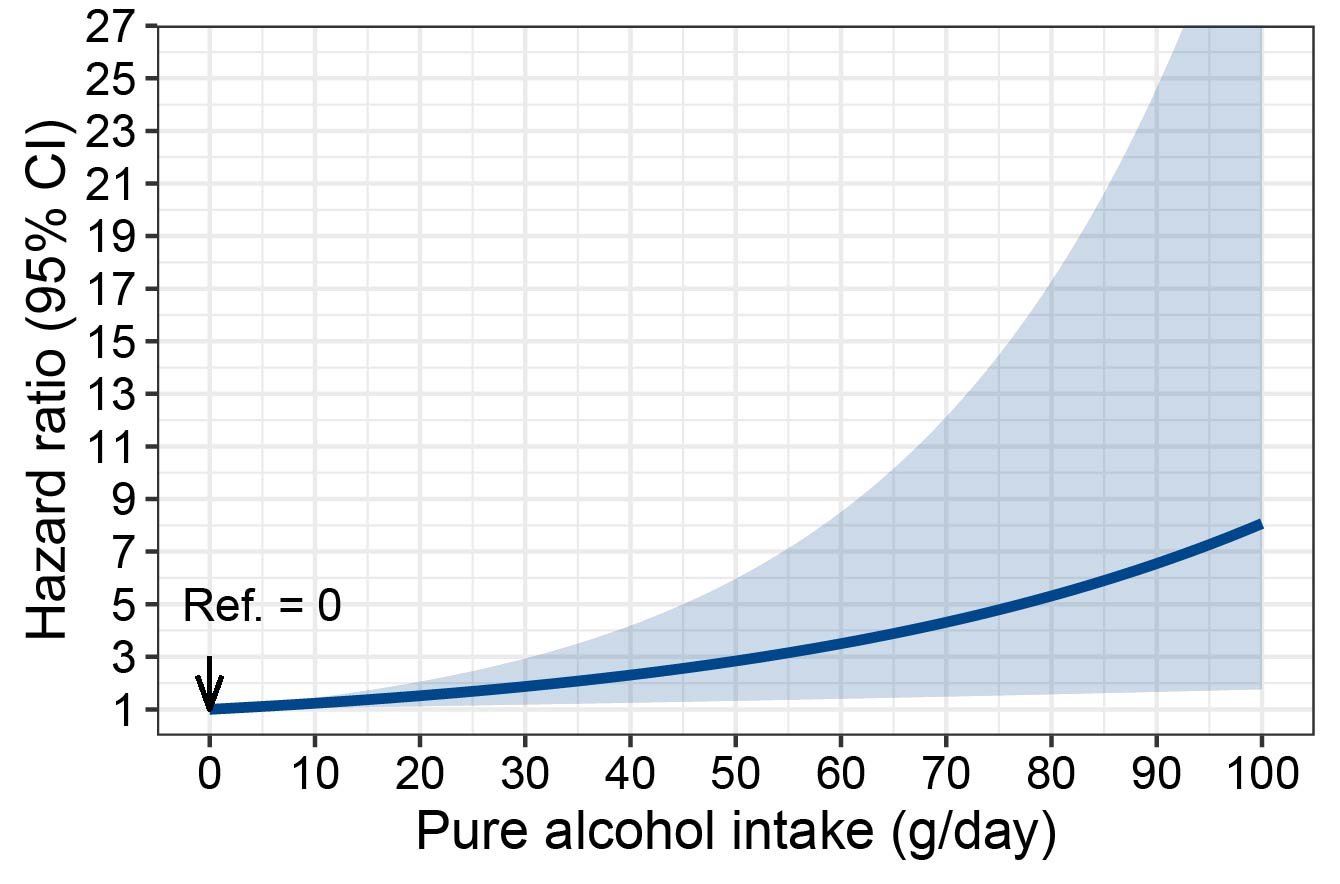


**Figure S15**. Association between alcohol intake and risk of hepatocellular carcinoma in individuals carrying CT/TT genotype of TM6SF2 rs58542926 and mainly drank wine.


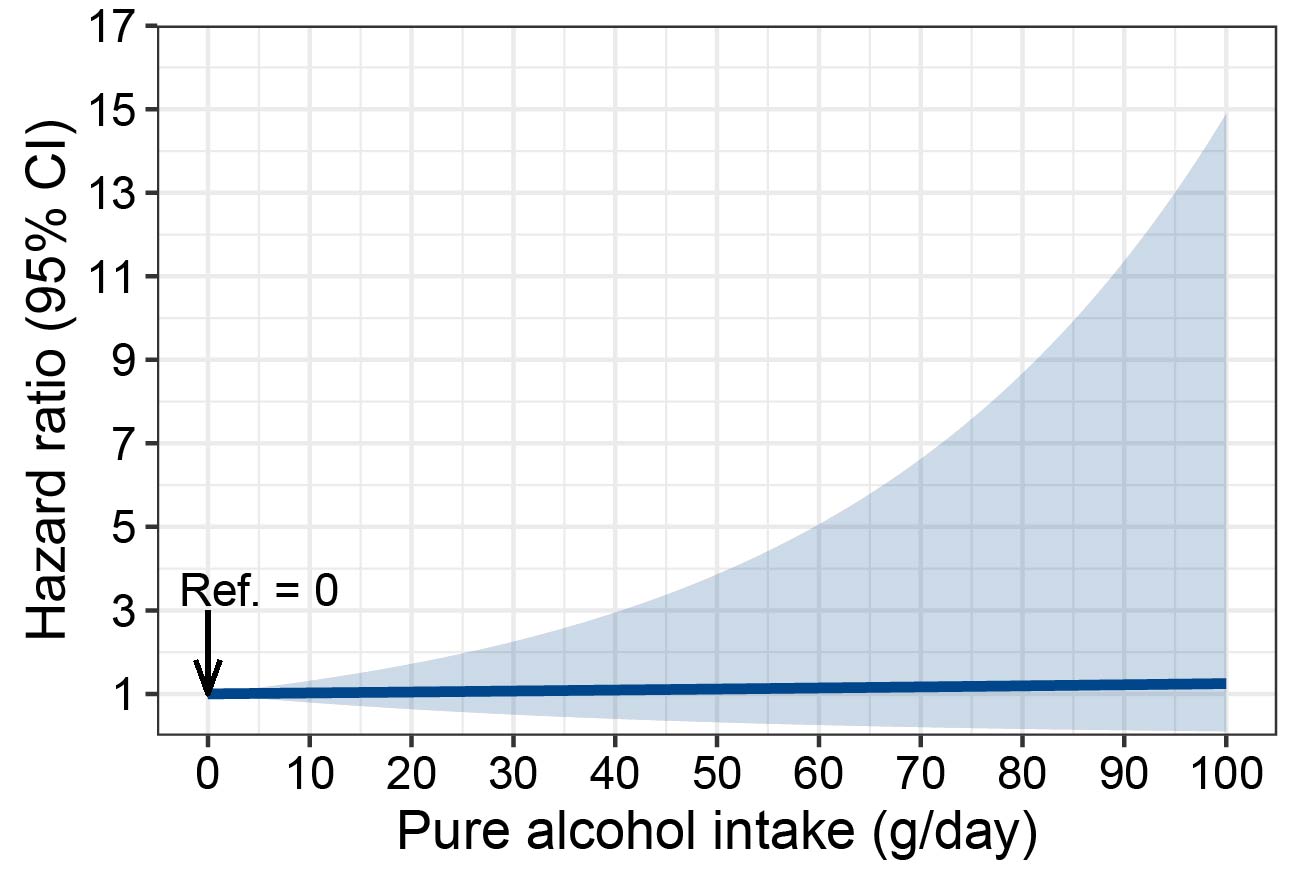


**Figure S16**. Effects of moderate drinking on serum levels of ALT and AST by sex.


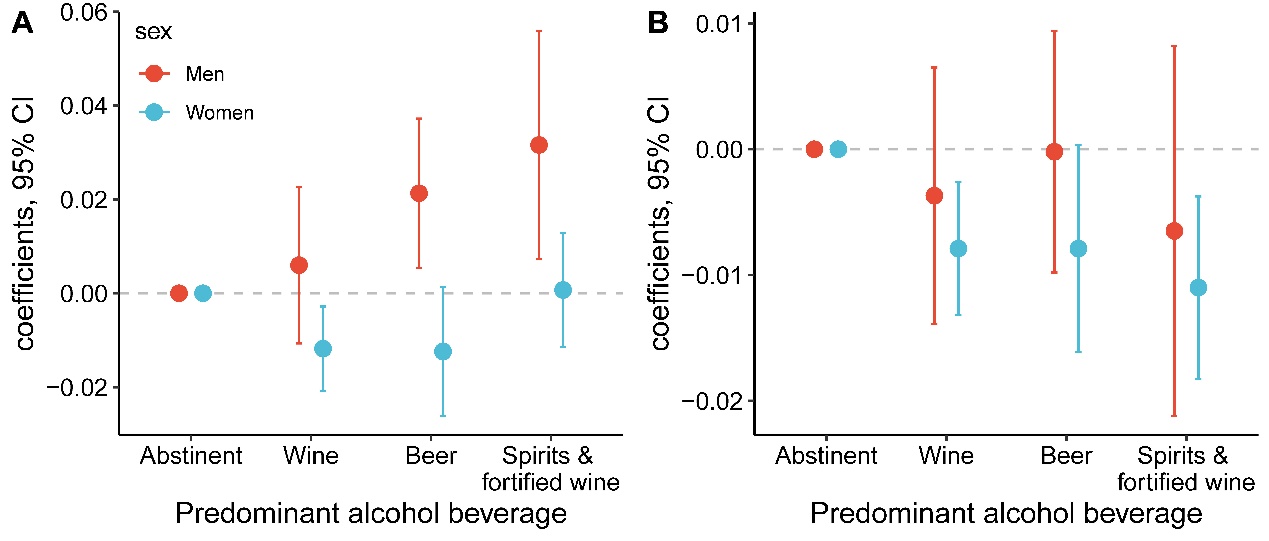


A, ALT; B, AST. The coefficients and 95% confidence intervals were obtained from linear regression model, in which log-transformed ALT or AST level was set as the response variable. Age was adjusted.

**Figure S17**. Effects of moderate drinking on serum levels of ALT and AST by age.


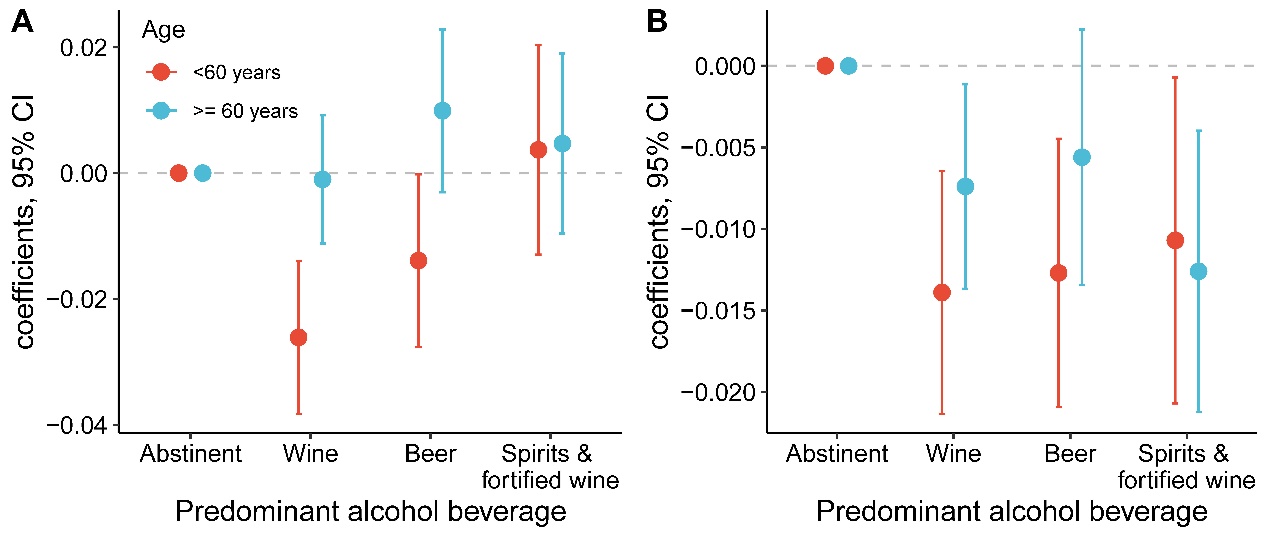


A, ALT; B, AST. The coefficients and 95% confidence intervals were obtained from linear regression model, in which log-transformed ALT or AST level was set as the response variable. Sex was adjusted.

**Figure S18**. Effects of moderate drinking on serum levels of ALT and AST by genotypes of TM6SF2 rs58542926.


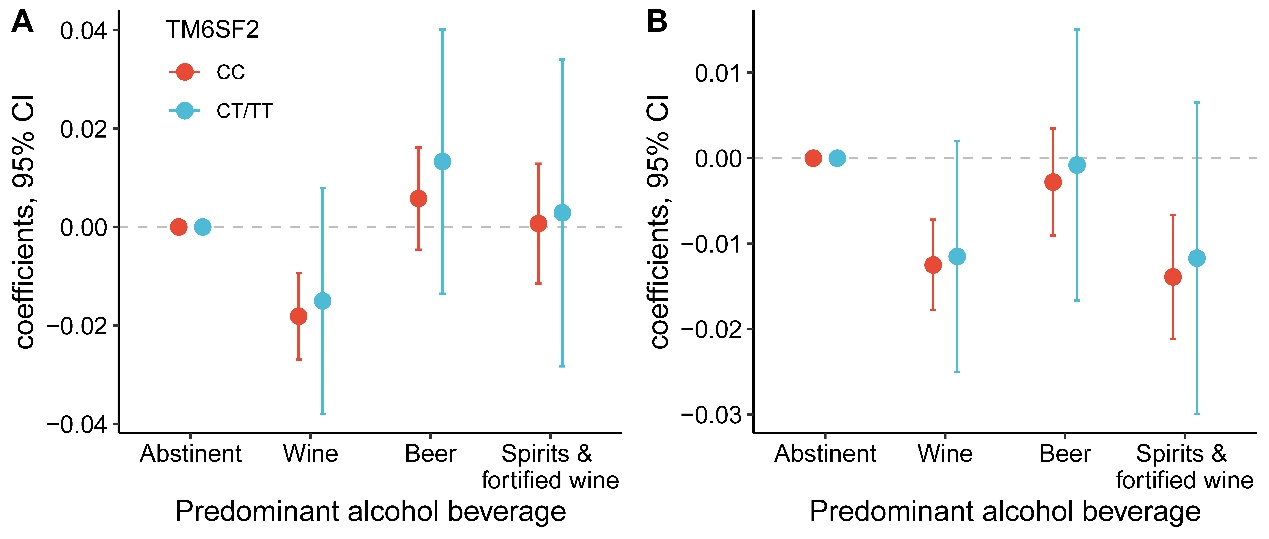


A, ALT; B, AST. The coefficients and 95% confidence intervals were obtained from linear regression model, in which log-transformed ALT or AST level was set as the response variable. Age and sex were adjusted.

**Figure S19**. Effects of moderate drinking on serum levels of ALT and AST by genotypes of PNPLA3 rs738409.


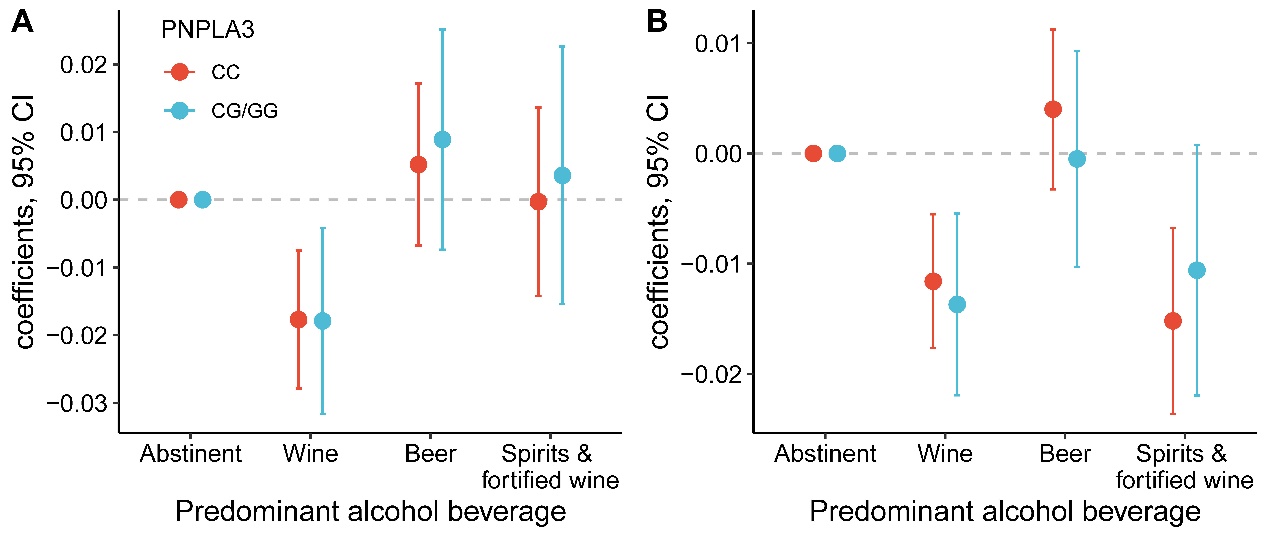


A, ALT; B, AST. The coefficients and 95% confidence intervals were obtained from linear regression model, in which log-transformed ALT or AST level was set as the response variable. Age and sex were adjusted.
