# Supplementary material for: Alcohol consumption and hepatocellular carcinoma: novel insights from a prospective cohort study and nonlinear Mendelian randomization analysis
Source: BMC Med. 2022 Oct 28;20:413. doi: 10.1186/s12916-022-02622-8 (PMC9615332; doi:10.1186/s12916-022-02622-8)
Supplement: Supplementary file 2 — Additional file 2: Table S1. Calculation of pure alcohol intake in the UK Biobank cohort. Table S2. Prevalent and incident diseases in the UK Biobank cohort. Table S3. Characteristics of the study participants by levels of alcohol drinking. Table S4. AIC values for the additive Cox regression models with different degree of freedom. Table S5. Statistics for the nonlinear Mendelian randomization analysis. Table S6. AIC values for the additive Cox regression models with the exclusion of people mainly drinking wine. Table S7. AIC values for the additive Cox regression models in people mainly drinking wine. [file 12916_2022_2622_MOESM2_ESM.docx]

Additional file 2: Tables S1-S7

Alcohol consumption and hepatocellular carcinoma: novel insights from a prospective cohort study and nonlinear Mendelian randomization analysis

Zhenqiu Liu, Ci Song, Chen Suo, Hong Fan, Tiejun Zhang, Li Jin, Xingdong Chen

**TABLE OF CONTENT**

[**Table S1.** Calculation of pure alcohol intake in the UK Biobank cohort. 2](#_Toc116377747)

[**Table S2**. Prevalent and incident diseases in the UK Biobank cohort. 3](#_Toc116377748)

[**Table S3.** Characteristics of the study participants by levels of alcohol drinking. 4](#_Toc116377749)

[**Table S4.** AIC values for the additive Cox regression models with different degree of freedom. 5](#_Toc116377750)

[**Table S5**. Statistics for the nonlinear Mendelian randomization analysis. 6](#_Toc116377751)

[**Table S6.** AIC values for the additive Cox regression models with the exclusion of people mainly drinking wine. 6](#_Toc116377752)

[**Table S7.** AIC values for the additive Cox regression models in people mainly drinking wine. 6](#_Toc116377753)

**Table S1.** Calculation of pure alcohol intake in the UK Biobank cohort.

|  | Red wine | White wine | Beer/cider | Spirits | Fortified wine | Others | Pure alcohol intake (g/day) |
| --- | --- | --- | --- | --- | --- | --- | --- |
| Type | Red wine | White wine/  champagne | Beer, bitter, lager, stout, ale, Guinness | Whisky, gin, rum, vodka, brandy | Sherry, port, vermouth | Alcopops etc. |  |
| Unit | Glass | Glass | Pint | Measure | Glass | Glass |  |
| Pure alcohol (g/100 ml) | 13 | 12 | 4.5 | 40 | 18 | 4 |  |
| Alcohol drinker status = Never (EID=20117) | / | / | / | / | / | / | 0 |
| Intake frequency (EID = 1558) | | |  |  |  |  |  |
| Special occasion only | EID= 4407;  1 glass = 125 ml | EID= 4418;  1 glass = 125 ml | EID= 4429;  1 pints = 568.3 ml | EID= 4440;  1 measure = 25 ml | EID= 4451;  1 glass = 75 ml | EID= 4462;  1 glass = 275 ml | (red wine×13/100×125+white wine×12/100×125+ beer×4.5/100×568.3+spirits×40/100×25+ fortified wine×18/100×75+other wine×4/100×275)/30 |
| 1-3 times/month |  |  |  |  |  |  |  |
| 1-2 times/week | EID= 1568;  1 glass = 125 ml | EID= 1578;  1 glass = 125 ml | EID= 1588;  1 pints = 568.3 ml | EID= 1598;  1 measure = 25 ml | EID= 1608;  1 glass = 75 ml | EID= 5364;  1 glass = 275 ml | (red wine×13/100×125+white wine×12/100×125+ beer×4.5/100×568.3+spirits×40/100×25+ fortified wine×18/100×75+other wine×4/100×275)/7 |
| 3-4 times/week |  |  |  |  |  |  |  |
| Daily or almost daily |  |  |  |  |  |  |  |

During a self-completed touchscreen interview taken at baseline appointment, participants were asked about their current drinking status (never, previous, current, prefer not to say) and were asked to report their average weekly and monthly alcohol consumption of a range of drink types (red wine, white wine/champagne, spirits, beer/cider, fortified wine, other wine). These questions were accompanied by pictures providing an example of a single measure of each drink type. From these measures we derived an average intake of alcohol consumption in gram per day. The volume of pure alcohol in each type of drink was referred to:

[*https://www.drinkaware.co.uk/facts/alcoholic-drinks-and-units/low-risk-drinking-guidelines#:~:text=UK%20alcohol%20unit%20guidance%3A%20CMOs%27%20Low%20Risk%20Drinking,and%20single%20occasion%20drinking.%20Low%20Risk%20Drinking%20Guidelines*](https://www.drinkaware.co.uk/facts/alcoholic-drinks-and-units/low-risk-drinking-guidelines#:~:text=UK%20alcohol%20unit%20guidance%3A%20CMOs%27%20Low%20Risk%20Drinking,and%20single%20occasion%20drinking.%20Low%20Risk%20Drinking%20Guidelines)

**Table S2**. Prevalent and incident diseases in the UK Biobank cohort.

| Diseases | ICD-10 codes for incident diseases | ICD-10 codes for prevalent diseases | ICD-9 codes for prevalent diseases |
| --- | --- | --- | --- |
| Liver diseases | C22.0 | B18.x, K70.0–K70.3, K70.9, K71.3–K71.5, K71.7, K73.x, K74.0-K74.2, K76.0, K76.2–K76.4, K76.8, K76.9, Z94.4, I85.0, I85.9, I86.4, I98.2, K70.4, K71.1, K72.1, K72.9, K76.5, K76.6, K76.7, K74.3-K74.6; C22.0-C22.9 | 571.2, 571.4-571.6, 456.0-456.21, 572.2-572.8 |
| Type 2 diabetes mellitus | / | E11.0-E11.9 | 250 |
| Cardiovascular diseases | / | I21.x, I22.x, I25.2, I09.9, I11.0, I13.0, I13.2, I25.5, I42.0, I42.5–I42.9, I43.x, I50.x, P29.0 | 410.x, 412.x; 428.x |
| Chronic kidney diseases | / | I12.0, I13.1, N03.2–N03.7, N05.2–N05.7, N18.x, N19.x, N25.0, Z49.0–Z49.2, Z94.0, Z99.2 | 582.x, 583-583.7, 585.x, 586.x, 588.x |
| Hypertension | / | I10-I15, O10 | 401.1, 401.9, 402.10, 402.90, 404.10, 404.90, 405.1, 405.9 |
| Dementia | / | F00-F03, F05.1, G30, G31.1 | 290.x |
| Rheumatic diseases | / | M05, M06, M31.5, M32-M34, M35.1, M35.3, M36.0 | 710.0, 710.1, 710.4, 714.0-714.2, 714.81, 725 |
| Cerebrovascular diseases | / | G45, G46, H34.0, I60-I69 | 430-438 |
| Cancers | / | C00.x–C26.x, C30.x–C34.x, C37.x–C41.x, C43.x, C45.x–C58.x, C60.x–C76.x, C81.x–C86.x, C88.x, C90.x–C97.x | 140.x-172.x, 174.x-195.8, 200.x-208.x |

The disease code was referred to *Medical Care; 2005;43(11):1130-1139.*

**Table S3.** Characteristics of the study participants by levels of alcohol drinking.

| Characteristics | Alcohol consumption level | | | | P for trend† |
| --- | --- | --- | --- | --- | --- |
|  | Abstinent | Low-to-moderate | Excess | Heavy |  |
| Sample size | 12,342 | 190,180 | 89,012 | 37,630 |  |
| Mean (SD) age (years) | 58.9 (8.0) | 56.8 (8.1) | 56.4 (7.9) | 56.1 (7.8) | <0.0001 |
| Female | 9332 (75.6) | 108,362 (57.0) | 40,566 (45.6) | 13,861 (36.8) | <0.0001 |
| Median (IQR) pure alcohol intake (g/day) | 0 | 12.2 (6.8-17.5) | 34.7 (27.9-43.2) | 71.2 (57.1-89.9) | <0.0001 |
| Dominated alcohol type§ |  |  |  |  |  |
| Wine | 0 | 51,895 (27.3) | 21,630 (24.3) | 8513 (22.6) | <0.0001 |
| Beer | 0 | 26,393 (13.9) | 15,048 (16.9) | 10054 (26.7) | <0.0001 |
| Spirits and fortified wine | 0 | 9179 (4.8) | 1756 (2.0) | 662 (1.8) | <0.0001 |
| Non-specific | 0 | 102,713 (54.0) | 50,578 (56.8) | 18401 (48.9) | <0.0001 |
| Smoking status |  |  |  |  |  |
| Never | 9945 (80.6) | 116,543 (61.3) | 41,000 (46.1) | 12,363 (32.9) | <0.0001 |
| Former | 1581 (12.8) | 59,846 (31.5) | 37,783 (42.4) | 17,424 (46.3) | <0.0001 |
| Current | 816 (6.6) | 13,791 (7.3) | 10,229 (11.5) | 7843 (20.8) | <0.0001 |
| Average household income |  |  |  |  |  |
| Less than 18,000 | 3632 (29.4) | 29,359 (15.4) | 11,559 (13.0) | 6019 (16.0) | <0.0001 |
| 18,000 to 30,999 | 2710 (22.0) | 40,962 (21.5) | 17,948 (20.2) | 8003 (21.3) | <0.0001 |
| 31,000 to 51,999 | 4707 (34.7) | 72,035 (37.9) | 32,201 (36.2) | 13,058 (34.7) | <0.0001 |
| 52,000 to 100,000 | 1041 (8.4) | 37,987 (20.0) | 20,680 (23.2) | 8121 (21.6) | <0.0001 |
| Greater than 100,000 | 252 (2.0) | 9837 (5.2) | 6624 (7.4) | 2429 (6.5) | <0.0001 |
| Physical activity |  |  |  |  |  |
| 0-1 day/week | 2446 (19.8) | 36,183 (19.0) | 16,653 (18.7) | 7683 (20.4) | 0.0023 |
| 2-4 days/week | 4906 (39.8) | 83,703 (44.0) | 38,710 (43.5) | 14,612 (38.8) | <0.0001 |
| 5-7 days/week | 4990 (40.4) | 70,294 (37.0) | 33,649 (37.8) | 15,335 (40.8) | <0.0001 |
| BMI category |  |  |  |  |  |
| <25 | 4268 (34.6) | 71,846 (37.8) | 30,157 (33.9) | 10,689 (28.4) | <0.0001 |
| 25-29.9 | 4693 (38.0) | 81,246 (42.7) | 41,067 (46.1) | 17,646 (46.9) | <0.0001 |
| >=30 | 3381 (27.4) | 37,088 (19.5) | 17,788 (20.0) | 9295 (24.7) | <0.0001 |
| *PNPLA3* rs738409* |  |  |  |  |  |
| CC | 7369 (61.5) | 113,645 (61.3) | 53,709 (61.8) | 22,716 (61.9) | 0.0078 |
| CG/GG | 4606 (38.5) | 71,847 (38.7) | 33,219 (38.2) | 13,990 (38.1) | 0.0078 |
| *TM6SF2* rs58542926 |  |  |  |  |  |
| CC | 10,260 (85.7) | 158,663 (85.5) | 74,321 (85.5) | 31,532 (85.9) | 0.2660 |
| CT/TT | 1715 (14.3) | 26,829 (14.5) | 12,607 (14.5) | 5174 (14.1) | 0.2660 |
| Mean ALT U/L (SD)^#^ | 2.97 (0.42) | 3.01 (0.43) | 3.06 (0.44) | 3.15 (0.47) | <0.0001 |
| Mean AST U/L (SD)^#^ | 3.20 (0.25) | 3.20 (0.25) | 3.23 (0.26) | 3.30 (0.31) | <0.0001 |
| HBV/HCV seropositivity*^,&^ | 3 (1.3) | 63 (1.8) | 39 (2.3) | 11 (1.5) | 0.6708 |

Values are numbers (percentage at column) unless stated otherwise.

*, 8063 individuals were missing on genetic data; 323,008 individuals were missing on HBV/HCV status.

§, The dominated beverage type of each participant was defined as the beverage contributing ≥75% to the total pure alcohol volume. Otherwise, no dominated type of beverage was assigned.

#, ALT and AST values have been log-transformed.

&, HBV seropositivity defined as both antigen HBc > 100 and antigen HBe > 150; HCV seropositivity defined as both antigen Core > 150 and antigen NS3 > 150.

†, Cochran-Armitage trend test was used for categorical variables; linear regression model was used for continuous variables. Alcohol intake level was set as 1-4 in the linear regression models.

Abbreviations: pys, person-years; BMI, body-mass index; SD, standard deviation; IQR, interquartile range; IRR, incidence rate ratio; CI, confidence interval. ALT alanine aminotransferase; AST, aspartate aminotransferase; HBV, hepatitis B virus; HCV, hepatitis C virus.

**Table S4.** AIC values for the additive Cox regression models with different degree of freedom.

|  | AIC values | | | | |
| --- | --- | --- | --- | --- | --- |
|  | df=1 | df=2 | df=3 | df=4 | df=5 |
| Overall | 4804.8 | 4804.9 | **4799.7** | 4799.8 | 4800.1 |
| Alcohol intake frequency |  |  |  |  |  |
| Less-frequent | 1489.8 | 1484.9 | **1486.8** | 1488.1 | 1489.7 |
| Frequent | 1433.9 | 1433.3 | 1435.1 | **1431.5** | 1432.4 |
| Regular | 1754.7 | 1756.6 | **1753.1** | 1755.3 | 1756.1 |
| Alcohol usually taken with meal | | |  |  |  |
| Yes | 1618.3 | **1617.3** | 1617.6 | 1619.0 | 1619.4 |
| No | 1274.4 | 1274.8 | **1274.3** | 1275.9 | 1277.7 |
| Alcohol beverage types |  |  |  |  |  |
| Wine | 984.2 | 983.2 | **982.4** | 982.9 | 984.0 |
| Spirits and fortified wine | **310.9** | 310.9 | 312.8 | 312.8 | 314.8 |
| Beer | **1194.8** | 1196.9 | 1198.8 | 1198.4 | 1200.2 |
| Sex |  |  |  |  |  |
| Men | **3461.0** | 3462.8 | 3461.5 | 3462.8 | 3464.3 |
| Women | 1124.0 | 1119.3 | **1117.3** | 1120.9 | 1123.1 |
| Age (years) |  |  |  |  |  |
| >=60 | **3405.6** | 3409.9 | 3408.2 | 3408.4 | 3407.9 |
| <60 | 1180.6 | 1177.1 | **1174.5** | 1176.6 | 1177.8 |
| ALT level |  |  |  |  |  |
| >=40 U/L | **1620.0** | 1621.7 | 1623.0 | 1624.9 | 1626.8 |
| <40 U/L | 2768.5 | 2768.8 | 2763.8 | 2763.3 | **2760.2** |
| PNPLA3 rs738409 |  |  |  |  |  |
| CC | 2259.5 | 2261.5 | 2254.3 | **2254.2** | 2254.7 |
| CG/GG | **2210.2** | 2210.9 | 2211.7 | 2213.0 | 2214.4 |
| TM6SF2 rs58542926 |  |  |  |  |  |
| CC | 3593.2 | 3594.1 | 3591.1 | **3590.1** | 3591.5 |
| CT/TT | **938.9** | 940.7 | 939.6 | 941.2 | 942.3 |

The blue bold numbers denote the lowest AIC value.

**Table S5**. Statistics for the nonlinear Mendelian randomization analysis.

| Estimate | Standard error | P value | Fractional polynomial degree p-value | Fractional polynomial non-linearity p-value | Quadratic p-value | Cochran Q p-value | P for trend |
| --- | --- | --- | --- | --- | --- | --- | --- |
| 7.04e-4 | 5.49e-4 | 0.199 | 0.855 | 0.386 | 0.383 | 0.499 | <0.0001 |

A low fractional polynomial non-linearity p-value indicates a non-linear relationship better fits the relationship than a linear model, and a low quadratic test p-value indicates a non-linear relationship between the exposure and the outcome. The Cochran Q p-value tests if LACE values differ more than would be expected by chance.

**Table S6.** AIC values for the additive Cox regression models with the exclusion of people mainly drinking wine.

|  | AIC values | | | | |
| --- | --- | --- | --- | --- | --- |
|  | df=1 | df=2 | df=3 | df=4 | df=5 |
| Women | **644.5** | 645.9 | 647.9 | 649.1 | 648.9 |
| Normal-ALT | **2143.6** | 2145.4 | 2145.2 | 2145.0 | 2145.7 |
| Age <60 years | **2819.0** | 2821.5 | 2820.4 | 2819.4 | 2819.0 |
| PNPLA3-CC | 1613.7 | 1615.4 | **1611.5** | 1612.9 | 1614.0 |
| TM6SF2-CC | **2814.5** | 2816.4 | 2815.3 | 2814.5 | 2816.4 |

The blue bold numbers denote the lowest AIC values.

**Table S7.** AIC values for the additive Cox regression models in people mainly drinking wine.

|  | AIC values | | | | |
| --- | --- | --- | --- | --- | --- |
|  | df=1 | df=2 | df=3 | df=4 | df=5 |
| Men | **392.5** | 394.5 | 396.2 | 398.4 | 340.2 |
| Abnormal ALT | **258.9** | 260.9 | 262.9 | 264.9 | 266.9 |
| Age>=60 | **611.7** | 613.0 | 612.5 | 614.3 | 616.4 |
| PNPLA3-CG/GG | **285.0** | 286.2 | 285.1 | 287.1 | 288.5 |
| TM6SF2-CT/TT | **206.8** | 207.3 | 208.7 | 210.1 | 211.8 |

The blue bold numbers denote the lowest AIC values.
