# Supplementary material for: Alcohol consumption and hepatocellular carcinoma: novel insights from a prospective cohort study and nonlinear Mendelian randomization analysis
Source: BMC Med. 2022 Oct 28;20:413. doi: 10.1186/s12916-022-02622-8 (PMC9615332; doi:10.1186/s12916-022-02622-8)
Supplement: Supplementary file 3 — Additional file 3: Method S1. Additive Cox regression model. Method S2. Nonlinear Mendelian randomization analysis. [file 12916_2022_2622_MOESM3_ESM.docx]

Additional file 3: Methods S1-2

Alcohol consumption and hepatocellular carcinoma: novel insights from a prospective cohort study and nonlinear Mendelian randomization analysis

Zhenqiu Liu, Ci Song, Chen Suo, Hong Fan, Tiejun Zhang, Li Jin, Xingdong Chen

**TABLE OF CONTENT**

[**Method S1:** additive Cox regression model 2](#_Toc116377889)

[**Method S2:** nonlinear Mendelian randomization analysis 3](#_Toc116377890)

**Method S1:** additive Cox regression model

Traditionally, the Cox model assumes that the hazard function can be written as:

$\alpha\left( t;Z \right)= \alpha_{0}(t)exp\left( \beta^{T}Z \right)$,

where 𝑡 is time, $Z={(Z_{1},\cdots,Z_{p})}^{T}$ is a 𝑝-dimensional vector of time-fixed covariates, 𝛽 is the associated vector of unknown regression parameters, and $\alpha_{0}\left( t \right)$ a nonnegative baseline hazard function. The effect of covariates estimated by any proportional hazards model can thus be reported as hazard ratios (HRs). The logarithm of the hazard ratio curve is then reduced to a straight line, indicating that the expected change in risk for a $(Z_{i}-z_{i,ref})$ change in 𝑍𝑖 is a constant value (the well-known proportional hazards assumption). However, there are many nonlinear associations existed between the outcome and covariates. One possible approach to incorporate nonlinear effects into the Cox model is to express the log hazard as an additive function:

$\alpha\left( t;Z \right)= \alpha_{0}(t)exp\left( \sum_{i=1}^{q} f_{i}\left( Z_{i} \right)+\sum_{i=q+1}^{p} \beta_{i}Z_{i} \right)$,

where the first 𝑞 covariates are continuous and introduced nonlinearly through (unknown) smooth functions, 𝑓𝑖, and the remaining ones are covariates introduced parametrically in the model.

The HR curve for a continuous predictor $Z_{i}$ in the additive Cox model can be written as:

$\mathrm{HR}\left( Z_{i},z_{i,ref} \right)=exp\left( f_{i}\left( Z_{i} \right)-f_{i}\left( z_{i,ref} \right) \right)$, where $z_{i,ref}$ means the specific value of the predictor taken as the reference. A natural estimate of the adjusted HR curve can be calculated as $\hat{{HR}_{i}}\left( Z_{i},z_{i,ref} \right)=exp\{\hat{f_{i}}\left( Z_{i} \right)-\hat{f_{i}}\left( z_{i,ref} \right)\}$ by replacing $f_{i}(\cdot)$ with the corresponding natural-spline estimate $\hat{f_{i}}(\cdot)$(or any other smoother).

**Method S2:** nonlinear Mendelian randomization analysis

The instrumental genetic tools for alcohol consumption were retrieved from a genome-wide association study (GWAS) of up to 1.2 million individuals (*Liu et al. Nat Genet. 2019;51(2):237-244*). In this GWAS, alcohol consumption was quantified by drinks per week (defined as the average number of drinks a participant reported drinking each week, aggregated across all types of alcohol). This phenotype was left-anchored at 1 and log-transformed prior to analysis, in order to prevent outliers from having undue leverage on analyses. A total of 941,280 subjects were included in the GWAS meta-analysis. Of note, to obviate winner-curse bias, we used GWAS summary data with the exclusion of UK Biobank participants that provided by the authors (Liu et al). The summary statistics of GWAS were available at <https://conservancy.umn.edu/handle/11299/201564>. We selected the SNP that reached GWAS significance (P = 5×10^-8^). To ensure the independence of genetic variables, we performed a clumping process (threshold of R^2^ was < 0.01, window size = 10 000 kb) using linkage disequilibrium (LD) estimates calculated from Europeans in 1000 Genomes project. Among those pairs of SNPs that had LD R^2^ > 0.01, we retained the SNP with the relatively lower P value. SNPs absent from the LD reference panel were also removed. The eligible SNPs shown in the following table were coded as 0, 1, and 2, and were summed to an allele score (AllS) for each participant in the UK Biobank cohort.

| RSID | CHR | Position | Effect allele | Other allele | P value |
| --- | --- | --- | --- | --- | --- |
| rs11940694 | 4 | 39414993 | G | A | 4.82E-14 |
| rs1229984 | 4 | 100239319 | C | T | 1.12E-65 |
| rs1302808 | 4 | 100291120 | A | C | 1.01E-9 |
| rs55872084 | 5 | 155902003 | T | G | 2.20E-8 |
| rs7187575 | 16 | 28990101 | T | C | 5.23E-9 |
| rs676388 | 19 | 49211969 | C | T | 2.31E-9 |

In nonlinear Mendelian randomization analysis, we first calculated instrument variable (IV) free alcohol intake (i.e., daily pure alcohol intake) by taking the residuals of the regression of alcohol consumption on the AllS (i.e., IV-free-Alcohol=Alcohol–β×AllS). We then divided the sample into 10 stratums by using residual alcohol intake. Then we calculated the linear mendelian randomization estimate, referred to as a localized average causal effect (LACE), in each stratum of the population as a ratio of coefficients: the association of the AllS with the outcome divided by the association of the AllS with the exposure. We performed meta-regression of the LACE estimates against the mean of the exposure in each stratum in a flexible semiparametric framework by using the derivative of fractional polynomial models of degrees 1 and 2. Two tests for non-linearity are reported: a trend test, which assesses for a linear trend among the LACE estimates, and a fractional polynomial test, which assesses whether a non-linear model fits the LACE estimates better than a linear model (Sun et al. BMJ. 2019; 364: l1042). This analysis was performed based on the *nlmr* R package (https://github.com/jrs95/nlmr).
